# CRISPR spacer profiling and prophage mining reveal diverse bacteriophages associated with *Streptococcus Mutans*

**DOI:** 10.1080/20002297.2026.2674332

**Published:** 2026-05-21

**Authors:** Xiaolin Chen, Mingrui Zhang, Liuchang Yang, Yuxing Chen, Yaqi Chi, Yiran Zhao, Zhe Ma, Yongliang Li, Xiaoyan Wang

**Affiliations:** a Department of Cariology and Endodontology, Peking University School and Hospital of Stomatology & National Center for Stomatology & National Clinical Research Center for Oral Diseases & National Engineering Research Center of Oral Biomaterials and Digital Medical Devices & Beijing Key Laboratory of Digital Stomatology & NHC Key Laboratory of Digital Stomatology & NMPA Key Laboratory for Dental Materials, Beijing, People's Republic of China; b School of Stomatology, Hebei Medical University, Shijiazhuang, People's Republic of China

**Keywords:** *Streptococcus mutans*, dental caries, phage therapy, CRISPR-Cas, spacer targeting, prophage, comparative genomics

## Abstract

**Background:**

Streptococcus mutans is a key cariogenic bacterium. Current antimicrobials lack species specificity, while phage-based approaches remain experimental and require more *S. mutans* phage isolates.

**Objective:**

To profile the diversity of *S. mutans*-associated phages and strain-level heterogeneity in phage exposure using genome-informed CRISPR spacer and prophage analyses.

**Materials and methods:**

We compiled 944 publicly available *S. mutans* genomes and dereplicated them into 735 non-redundant strains. CRISPR–Cas systems, spacers, spacer targets, and putative prophages were identified, quality-assessed, and functionally annotated. Phylogenetic relationships of (pro)phages were evaluated using terminase large subunit proteins, and comparative genomics compared spacer-positive and spacer-negative strains.

**Results:**

CRISPR systems were detected in 548/735 strains, yielding 14,263 spacers, 1,864 phage-targeting spacers mapped to 110 viral genomes, including 41 cultured isolates, 51 metagenome-assembled phages, and 18 uncultured viral genomes. The most frequently targeted cultured phage was phiKSM96, whereas metagenome-assembled *Caudoviricetes* ctNo011 showed broader targeting. Prophage mining identified 186 regions in 130 strains, including 37 of ≥ medium quality and elements related to ctNo011 and phiKSM96. TerL phylogeny showed that most high-quality endogenous prophages clustered with phiKSM96 and ctNo011.

**Conclusion:**

These findings reveal a vast, uncultivated phage repertoire targeting *S. mutans*, providing a critical genomic roadmap to guide the future isolation of novel phages for caries prevention.

## Introduction

Dental caries is a highly prevalent, multifactorial chronic infectious disease worldwide, posing a significant threat to human oral health and quality of life. Within the complex oral microbiome, dental caries is increasingly viewed as an ecological dysbiosis driven by localized biofilm acidification and polymicrobial interactions [1–3]. Rather than acting in isolation, the primary cariogenic pathogen *Streptococcus mutans* cooperates with other pathobionts to assemble highly organized 3D spatial superstructures [4]. Within these structured microenvironments, the outstanding ability of acidogenicity and aciduricity make *S. mutans* outcompete commensals and drive continuous enamel demineralization [5,6]. Furthermore, *S. mutans* exhibits remarkable genomic and phenotypic diversity across different strains [7]. This extensive intraspecies heterogeneity, largely shaped by horizontal gene transfer and mobile genetic elements, drives variations in strain-specific features, such as cell-surface structures and adaptive immune repertoires. Understanding this strain-level diversity is critical, as it dictates how different *S. mutans* populations interact with and adapt to exogenous genetic invasions within the oral ecosystem.

Therefore, the key to clinical caries management is controlling the abundance of *Streptococcus mutans*. Current clinical approaches include mechanical biofilm removal and dietary interventions. In addition, the use of chemical agents for plaque removal is also a potential means of caries prevention. For example, fluoride is an important drug for caries prevention, which not only promotes enamel remineralization but also inhibits the growth of various bacteria, including *S. mutans* [8]. Chlorhexidine is a broad-spectrum antimicrobial agent. Chlorhexidine mouthrinse is considered the ‘gold standard’ for controlling oral plaque biofilm and is often used for caries prevention in special populations [9,10]. However, long-term use can lead to tooth staining, taste alteration, and disruption of the microbiome balance. A significant drawback of these therapies is their lack of species-specificity; while targeting *S. mutans*, they simultaneously harm commensal microbes known to defend against pathogenic biofilm colonization. To address this, experimental microbiome-targeted strategies, such as precision-guided antimicrobial peptides [11], are being actively explored. Along this line of species-specific targeting, harnessing natural biological agents like bacteriophages has also emerged as a vital frontier in dentistry [12].

Therapy based on bacteriophages offers a conceptually attractive alternative due to minimal impact on commensal microbiota. However, due to the narrow host ranges of individual phages and the rapid evolution of bacterial resistance, it is difficult to achieve effective pathogen clearance via single-phage treatments [13]. Therefore, therapeutic cocktail mixed with multiple bacteriophages, which shows lower resistance onset and enhanced bacterial clearance, has been proposed and explored in various experimental models [13]. Establishing and developing the phage therapy targeted to *S. mutans* has garnered growing attention as a novel strategy for caries prevention. However, there is a fundamental obstacle to designing these experimental phage mixtures to *S. mutans,* which is that only 5 phages have been isolated and sequenced: M102 [14], M102AD [15], smHBZ8 [16], ϕAPCM01 [17], and phiKSM96 [18]. This limitation severely hampers efforts to design combinatorial phage therapies. It remains unclear how many distinct phages exist in the oral cavity that can effectively target this species.

CRISPR-Cas (Clustered Regularly Interspaced Short Palindromic Repeats and CRISPR-associated proteins) systems constitute an adaptive immune mechanism in bacteria, enabling these organisms to detect and neutralize foreign genetic elements such as phages. Upon encountering foreign DNA, the host can integrate short fragments, known as spacers, into its CRISPR loci, forming a molecular ‘memory’ of past invasions [19]. This unique property positions CRISPR arrays as historical records of exogenous DNA invasion, providing valuable insights into the diversity and ecological relevance of past phage invasion [19]. Classified by their signature Cas effectors, the adaptive immune repertoire relies predominantly on Type II (e.g. II-A/II-C; single Cas9) and Type I systems (e.g. I-C/I-E; multi-protein Cascades). These distinct architectures likely confer different efficiencies and specificities in capturing viral sequences. Previous studies have demonstrated the feasibility of using spacer sequences to trace unknown phage lineages, identify broad patterns of phage-bacteria co-evolution, and even uncover novel viruses from metagenomic databases [20]. While recent profiling of *S. mutans* spacers has provided insights into their interactions with mobile genetic elements [21], the vast majority of its targeted 'viral dark matter' remains uncharacterized.

Beyond CRISPR records, bacteriophages can directly integrate as prophages, acting as critical drivers of genomic diversification and pathogenicity in *S. mutans* [22]. Despite their potential significance, the *S. mutans* prophage landscape remains poorly characterized, with only limited systematic investigation in public genomes and clinical isolates [22,23]. The acquisition of these mobile genetic elements is profoundly influenced by HGT, particularly through natural competence. Interestingly, while natural competence in taxa like *Neisseriaceae* and *Pasteurellaceae* relies on highly specific DNA uptake sequences that restrictively shape prophage acquisition [24–26], *S. mutans* lacks such strict sequence barriers [27]. As a highly competent species in dense oral biofilms, *S. mutans* may therefore acquire a broader diversity of phage-derived genetic material. However, much like the spacer-targeted 'viral dark matter', the full extent of prophage diversity and the functional cargo integrated within large-scale *S. mutans* populations remain to be systematically elucidated.

Thus, in the present study, to clarify the diversity of phages targeting *S. mutans*, we deeply analyzed CRISPR spacer sequences in *S. mutans* genomes. In parallel, we performed prophage prediction to identify integrated temperate phages across diverse strains. By integrating these datasets, we aimed to gain preliminary insights into the diversity of phages associated with *S. mutans* and offer a framework for guiding future efforts in phage therapy.

## Material and methods

### Genomic data

A total of 944 *Streptococcus mutans* genomes were included in this study. Of these, 477 assemblies were obtained as assembled genomes from the Shields laboratory dataset (provided in *.*gbk format) [21]. In addition, 385 genome assemblies were retrieved from the Human Oral Microbiome Database (HOMD) [28], 57 were obtained from the dataset reported by Omar E. Cornejo [29], and 25 were obtained from Merve Yildirim Ucuncu [30] via GenBank/NCBI. These public datasets included complete genomes, scaffolds, and contig-level assemblies; to maximize strain representation, no additional exclusion was applied based on assembly level. All genomes were subjected to pairwise average nucleotide identity (ANI) analysis using FastANI v1.34 [31]. Genomes sharing 100% ANI with >95% alignment coverage were considered to represent the same strain. Based on these criteria, 735 non-redundant *S. mutans* strains were retained for downstream analyses.

### Phylogenetic analysis

Core genome SNPs were identified using Snippy v4.6.0 with default parameters, using a representative *S. mutans* genome (SMU665) as the reference. Core genome alignments were generated using Snippy-core and used for phylogenetic inference with IQ-TREE v2.2.2 [32]. A maximum-likelihood tree was reconstructed under the GTR substitution model (DNA mode) with 1,000 ultrafast bootstrap replicates. Pairwise evolutionary distances were computed from the core SNP alignment using IQ-TREE. Tree visualization was performed using iTOL v6 [33].

### CRISPR spacer identification and target analysis

CRISPR-Cas systems in the 735 non-redundant *Streptococcus mutans* genomes were identified using the CRISPRCasTyper (cctyper) v1.8.0 with default settings [34]. Predicted spacer sequences were extracted and compiled, and each spacer was annotated with its corresponding isolate name, contig ID, and spacer index. Spacer target identification was performed using BLASTn v2.5.0 [35] against reference sequences of phages, integrative and conjugative elements (ICEs), fungi, archaea, and bacteria. Viral, archaeal, and fungal reference genomes were retrieved from the NCBI RefSeq database and Cenote Human Virome Database (CHVD) [36]. Uncultivated Viral Genomes (UViGs) were sourced from the entire dataset of 14,367 high-confidence *Streptococcus*-associated viral genomes in the IMG/VR database [37]. Additionally, ICE sequences were obtained from the ICEberg 2.0 database (https://bioinfomml.sjtu.edu.cn/ICEberg2/download.html) [38]. BLASTn searches were conducted using the megablast task with a seed length of 28 bp. Hits were retained for downstream analyses using a threshold of E-value ≤ 1 × 10^−3^, with minimum percent identity ≥ 90 and maximum mismatches ≤ 2. Data processing and visualization were performed in R v4.3.0 using the pheatmap package.

### Prophage identification and functional annotation

Putative prophages in *Streptococcus mutans* genomes were identified using VIBRANT v1.2.0 [39]. VIBRANT was run with default parameters, and prophage quality was assessed using CheckV v1.0.3 [40]. All candidate prophage regions were included for prevalence statistics and taxonomic alignment, whereas only prophages classified as medium quality or higher (i.e. ≥50% completeness, ensuring the retention of core viral modules) were carried forward for visualization and high-confidence presentation.

Predicted prophage proteins were annotated using multiple databases. Carbohydrate-active enzymes were identified using dbCAN3 [41]. Virulence factors were annotated using VFDB [42], and antimicrobial resistance genes were detected using CARD [43]. Host defense and anti-defense genes were identified using PADLOC-DB v2.0.0 [44] and dbAPIS [45], respectively. Phage structural and lifecycle-related proteins were annotated using VOG [46], with supplementary domain annotation performed using Pfam-A [47].

Prophage taxonomic affiliation was inferred by BLASTn (v2.5.0) against the NCBI viral RefSeq database (E-value ≤ 1e−5). Hits were filtered by percent identity (pident ≥ 90) and query coverage (qcovs ≥ 40). Filtered alignments were merged on each query into continuous, non-overlapping segments based on query-coordinate overlap. For each segment, hits were aggregated by subject accession, and the best-supported assignment was taken as the accession with the highest summed bit score; segment coordinates, coverage of the selected accession within the segment, and summary alignment statistics were recorded.

### Phylogenetic analysis of bacteriophages and prophages

Phylogenetic analysis was performed using terminase large subunit (TerL) proteins from representative *Streptococcus* phages, highly targeted UViGs, and prophages identified in this study [48]. Putative TerL homologs were identified by BLASTp against a curated reference set (E-value < 1e-5). Amino acid sequences were aligned with MAFFT v7.526 using the L-INS-i algorithm (−localpair −maxiterate 1000) [49]. The alignment was then trimmed with trimAl v1.4 using -automated1 [50]. A maximum-likelihood (ML) phylogenetic tree was reconstructed with IQ-TREE v3.0.1 [51]. ModelFinder (-m TEST), 1,000 ultrafast bootstraps (-bb 1000), and 1,000 SH-aLRT replicates (-alrt 1000) were applied. Trees were visualized and annotated in iTOL v6 [33].

### Comparative genome analysis

All 735 non-redundant *S. mutans* genomes were re-annotated using Bakta [52] to avoid inconsistencies arising from different annotation schemes. The domain information and KEGG orthology were annotated using eggNOG-mapper v2.1.12 [53]. For comparative analyses, strains were grouped based on phage-specific CRISPR spacer evidence (spacer-positive vs spacer-negative), and subgroup-specific genes (or KO terms) were identified accordingly. Enrichment analysis was performed in R using ClusterProfiler to map candidate gene sets to KEGG pathways, and dot plots were generated to visualize shared pathways (adjusted *p* < 0.05).

### Statistical analysis

Statistical analyses were conducted in R v4.3.0 using the rstatix package. Due to non-normal data distributions, non-parametric methods were utilized. The Wilcoxon rank-sum and Kruskal-Wallis tests (with Dunn’s post-hoc and Bonferroni correction) were used for group comparisons across phylogenetic clades and CRISPR subtypes. Spearman's rank correlation was applied to assess associations among continuous and ordinal genomic features (e.g. CRISPR array counts, spacer abundance, targeting breadth, and prophage quality).

## Results

### CRISPR-Cas systems are widespread in *S. mutans*

**Figure 1.**
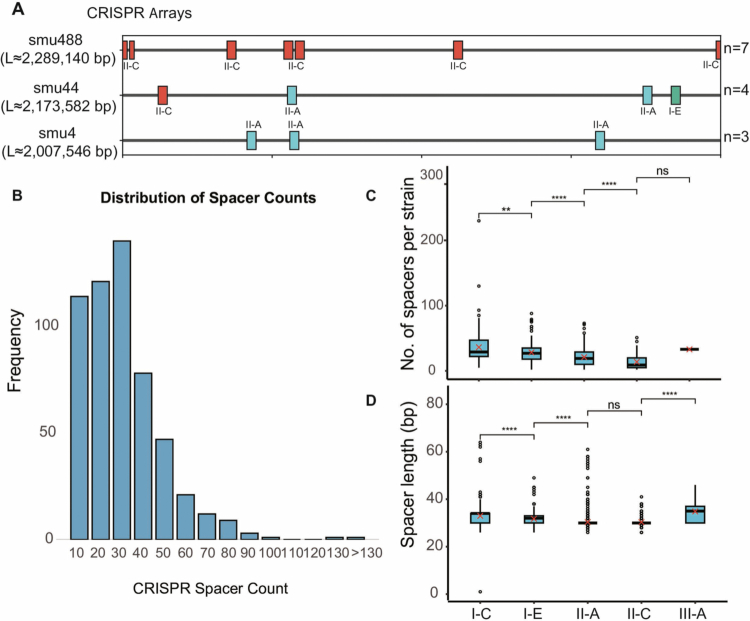
CRISPR array and spacer features across *Streptococcus mutans* genomes. (A) Genomic locations of CRISPR arrays in randomly selected representative strains are shown along relative genome coordinates (0-1); colours indicate CRISPR-Cas subtypes and *n* denotes the number of arrays. (B) Frequency distribution of total CRISPR spacer counts per strain. (C) Spacer counts per strain by subtype. In boxplots, centre lines indicate medians, boxes the interquartile range(IQR), whiskers 1.5 × IQR, and red crosses means. (D) Spacer length distributions by CRISPR-Cas subtype.

To investigate the prevalence of bacteriophages in *S. mutans*, we initially collected genomic information of 735 *S. mutans* strains from public databases, and all strains were renumbered as SMU1-SMU735 (Table S1) for the downstream analysis. Among these strains, 548 were found to carry potential CRISPR systems. Of these, 257 strains contained only one CRISPR system, 218 strains harbored two CRISPR systems, and 73 strains possessed between three and seven CRISPR systems (Figure 1A). However, based on SNP-based phylogenetic analysis, no clear correlation was observed between the number of CRISPR systems and strain evolution. According to CRISPR system typing, the most common type was Type II-A, accounting for approximately 37%. This was followed by types I-C, I-E, and II-C (Table S2).

Spacer sequences within CRISPR arrays serve as important evidence of foreign DNA invasion. We next characterized the spacer counts in *S. mutans* and found pronounced heterogeneity across CRISPR-containing strains, ranging from 1 to 130 spacers per genome (median: 21.1; interquartile range: 11-32) (Figure 1B). The number of spacers exhibits notable differences among CRISPR types. The I-C subtype contained the highest average number of spacers (36.2 ± 26.1), while the II-C subtype had the lowest average (13.8 ± 11.9) (Figure 1C). Although spacer quantities varied significantly, no substantial difference was observed in spacer lengths across different types (Figure 1D).

### The original derivation of CRISPR spacers

**Figure 2.**
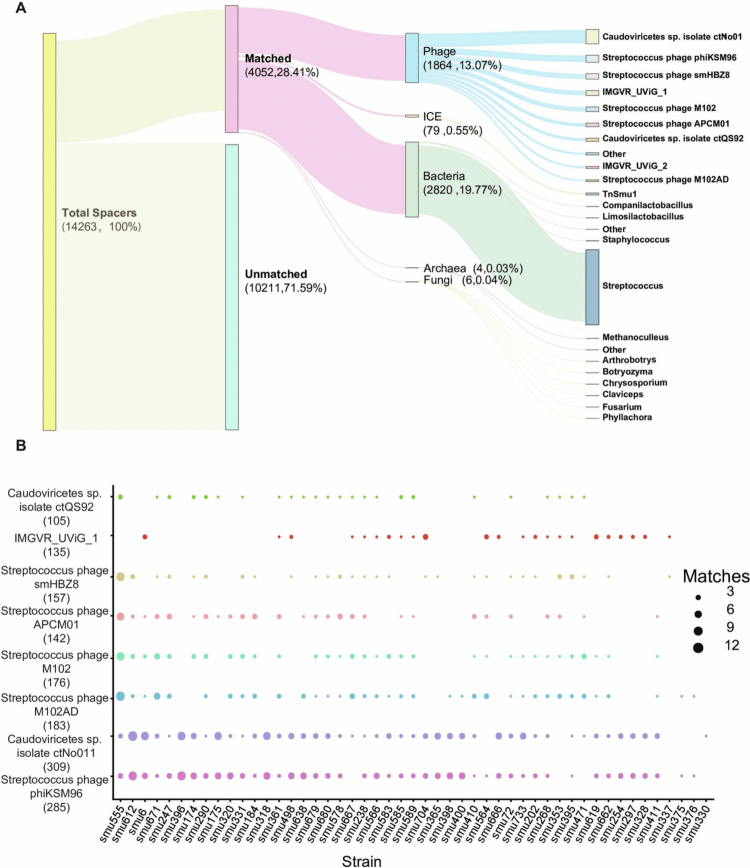
CRISPR spacer mapping and spacer-phage match patterns in *Streptococcus mutans*. (A) Unique spacers classified as matched or unmatched and assigned to target categories (phage, ICE, bacteria, archaea, and fungi); within each category, the most frequent targets are shown and the remainder grouped as ‘Other’. (B) Spacer-phage matches across strains and phages; point size indicates the number of spacers per strain-phage pair after filtering low-abundance rows/columns. The number in parentheses indicates the number of matched strains.

To delineate the exogenous selective pressures that have shaped the CRISPR-Cas immune repertoire of *S. mutans*, a comprehensive mapping of all detected 14,263 spacers to known foreign genetic elements was performed. In total, 3,741 spacers (26.23%) produced at least one significant match, whereas 10,522 spacers (73.77%) had no identifiable targets. Since a single spacer can yield multiple alignments and can match different target categories (e.g. prophage-derived sequences embedded in bacterial chromosomes), we observed 4,462 spacer-target hit events in total, including 1,553 phage-targeting hits and 2,820 hits to bacterial genomic elements; thus, category counts are not mutually exclusive. The remaining 89 hit events matched fragments such as ICE, archaea, and fungal genomes (Figure 2A).

Next, we conducted an in-depth analysis of the matched events between spacers and bacteriophages in NCBI and CHVD databases. A total of 1,553 spacers were found to match 92 bacteriophages, among which 41 have been isolated and genome-sequenced (Table S3). Notably, the five most frequently matched phages correspond to the previously reported *S. mutans* phages phiKSM96, M102AD, M102, smHBZ8, and ϕAPCM01 (Figure 2B).

PhiKSM96 was the most frequently targeted phage, with 562 spacers identified from 285 strains. Meanwhile, ϕAPCM01 matched spacers from 142 strains. The remaining 36 phages matched a smaller number of strains (Table S3, Figure 2B). Beyond these isolated sequenced phages, we identified 51 additional bacteriophages assembled from metagenomic sequencing data. Among these, the *Caudoviricetes* species ctNo011 was matched by 567 spacers from 309 strains, exceeding the number of strains matched by phiKSM96. Another notable phage, *Caudoviricetes* ctQS92, was also identified, with corresponding spacers observed in 105 strains (Figure 2B). The 42.07 kb genome of ctNo011 (62 ORFs) encodes typical *Caudoviricetes* modules for DNA packaging, structural assembly, and host lysis. Crucially, it harbors a putative integrase, providing direct functional evidence that ctNo011 operates as a temperate phage. Similarly, while the 12.44 kb sequence of ctQS92 represents a partial contig, it strictly conserves essential morphogenetic modules, including structural capsid and tail components.

To further explore the remaining 10,522 uncharacterized spacers, we queried them against the IMG/VR database. This analysis successfully matched an additional 311 distinct spacers to 18 highly confident uncultivated *Streptococcus* viral genomes (UViGs), five of which are explicitly predicted to infect *S. mutans* (Table S3). Notably, the most frequently targeted genome, IMGVR_UViG_1 (MGVR_UViG_2558860320_000002), was matched by 188 distinct spacers distributed across 148 strains.

Next, we performed a comparative genomic analysis on these strains to investigate whether strains carrying different phage spacers exhibit different genome components. *S. mutans* genomes were categorized into two groups based on phage-specific CRISPR spacer evidence: a spacer-positive group and a spacer-negative group. The spacer-positive group contained 335 unique genes, while the spacer-negative group contained 606 unique genes (Figure S1A). To further resolve strain distribution patterns within the spacer-positive group, strains were subdivided into subgroups according to the specific phage targeted by spacers in each strain (i.e. ϕAPCM01, M102, M102AD, phiKSM96, smHBZ8). We found that M102, phiKSM96, and smHBZ8 subgroups harbored 1, 46, and 53 distinct unique genes, respectively, while no specific genes were detected in the ϕAPCM01 or M102AD subgroups under our criteria (Figure S1B). The unique genes for each group are summarized in Table S4.

### The prediction of prophages and their function in *S. mutans*

**Figure 3.**
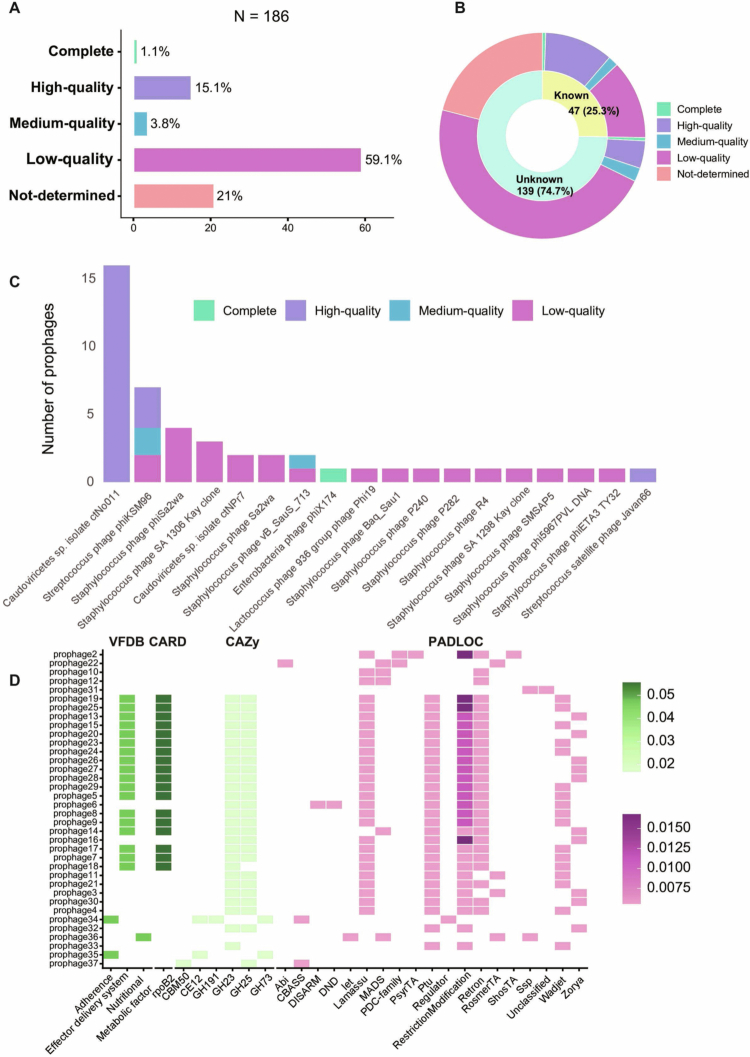
Prophage quality, predicted sources, and functional profiles in *S. mutans*. (A) CheckV quality distribution of predicted prophage regions. (B) Prophages assigned to a predicted source (‘Known’) versus unassigned (‘Unknown’), with CheckV quality proportions shown for each group. (C) Predicted sources of ‘Known’ prophages with CheckV quality stratification. (D) Functional annotations across VFDB, CARD, CAZy and PADLOC (proportions standardised within each database; prophages with CheckV ≥ Medium-quality).

Across 735 *S. mutans* genomes, 186 putative prophage regions were identified (distributed across 130 unique strains), corresponding to an overall prophage prevalence of 17.7% (Table S5). Among these strains, 107 (82.3%) carried one prophage, 16 carried two, and a small number carried three or more; one strain was predicted to encode up to 20 prophage regions. According to the quality examination of CheckV, we classified the 186 prophage fragments into 5 distinct quality groups based on their estimated completeness: Complete, High-quality, Medium-quality, Low-quality, and Not-determined. Two fragments were identified as complete prophages (Figure 3A); however, prophage1 showed a short contig length, which is not reasonable for a complete genome, so we excluded it manually for the next analysis. ‘High-quality’ and ‘Complete’ elements exhibited 100% completeness in our dataset. Taxonomic alignment linked 47 prophage regions to known taxa (Figure 3C): 16 regions matched *Caudoviricetes* sp. ctNo011, 7 matched *Streptococcus* phage phiKSM96, 19 matched *Staphylococcus* phages (e.g. vB_SauS_713), and single regions matched *Streptococcus* satellite phage Javan66, *Lactococcus* phage Phi19, and *Enterobacteria* phage phiX174.

Functional annotations revealed diverse encoded potential across prophage regions: 69 carbohydrate-active enzyme (CAZy) family hits (across 36 prophages, dominated by glycoside hydrolases including GH23 and GH25, alongside carbohydrate-binding modules (CBM13, CBM50) and glycosyltransferases such as GT2), 42 virulence factor (VFDB) hits (across 28 prophages, including ‘Effector delivery system’ and ‘Exotoxin’), 19 antimicrobial resistance (CARD) hits (across 19 prophages, all encoding ‘rpoB2’), 484 defense system (PADLOC) hits (encompassing 22 system types, with the ‘Restriction Modification’ and ‘Retron’ as the most prevalent), and 2,289 Virus Orthologous Group (VOG) hits (covering core lifecycle modules such as structural components, packaging, replication/nucleotide metabolism, integration/lysogeny, lysis/host interaction, and regulation) (Figure 3D).

### Phylogenetic analysis of *S. mutans* reveals correlations between CRISPR-Cas systems, phage targeting, and prophage carriage

**Figure 4.**
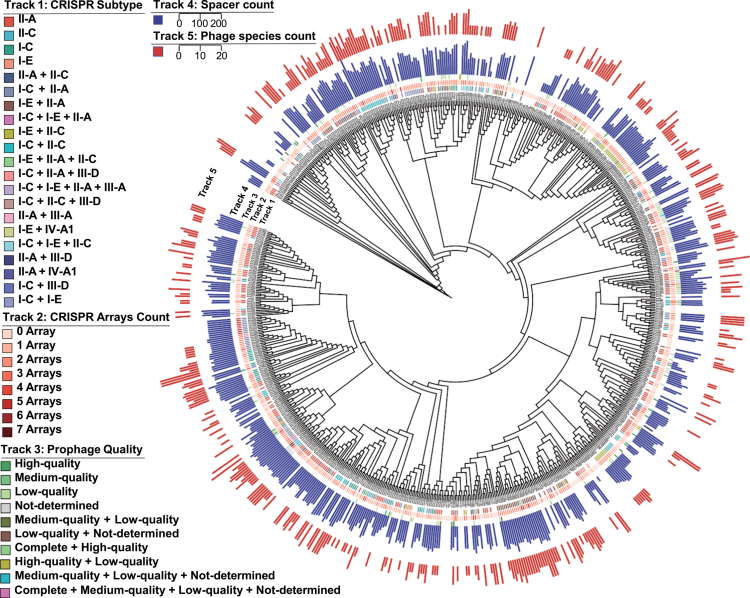
Phylogenetic tree of *S. mutans* strains and their corresponding CRISPR and prophage characteristics. From the inner to the outer tracks, the rings indicate CRISPR subtype, CRISPR arrays count per genome, prophage quality profile, log-transformed spacer count, and the log-transformed number of targeted phage species. Colors in the CRISPR subtype track represent different subtype compositions, whereas colors in the prophage quality track denote either single or combined prophage quality categories present in each genome.

Having characterized CRISPR-Cas immunity and endogenous prophages separately, we next examined their phylogenetic distribution and potential interactions across the *S. mutans* population. Mapping CRISPR-Cas features and prophage data onto the core-genome phylogeny provided a comprehensive view of their distributions (Figure 4).

We first assessed the overall features of CRISPR-Cas systems and their relationship with phage targeting. Array counts correlated strongly with spacer counts (Spearman's *ρ* = 0.771, *p* < 0.001) and with the number of targeted phages (*ρ* = 0.630, *p* < 0.001). Spacer counts also strongly predicted targeting counts (*ρ* = 0.819, *p* < 0.001). Moreover, lineages carrying complex, multi-subtype systems (e.g. I-C + I-E + II-A) had significantly more arrays and targeted more phages than those with single subtypes (Kruskal-Wallis, *p* < 0.001).

We next performed a more detailed analysis to explore how specific CRISPR subtypes correspond to particular phages. At the strain level, we examined the CRISPR subtype composition of strains carrying spacers that target different phages. The subtype composition shifted stepwise depending on the phage in question. For spacers targeting ϕAPCM01, smHBZ8, and M102, subtype II-A predominated (~48–54%), with I-E accounting for ~13–18%. For spacers targeting ctNo011, phiKSM96, and M102AD, the proportion of II-A decreased to ~41–44%, whereas I-E increased to ~21–26%. Notably, for ctQS92, II-A further dropped to ~29%, and I E became the most prevalent subtype (~43%) (Figure S2). At the array level, given that multiple CRISPR subtypes can coexist within a single strain, we traced each spacer back to its originating array subtype. The results were consistent with the strain-level analysis. Spacers targeting ϕAPCM01, smHBZ8, M102, and M102AD were predominantly acquired by subtype II-A (~43−50%), with a smaller contribution from I-E (~9–20%). In contrast, spacers targeting ctNo011, phiKSM96, and ctQS92 showed a more balanced subtype composition: the II-A proportion dropped to ~31−38%, and type I subtypes (I-E and I-C) each contributed more substantially, ranging from ~22% to ~32% depending on the phage (Figure S3).

Next, we examined whether CRISPR-Cas system is associated with endogenous prophage integration. The results show that prophage-positive and prophage-negative strains did not differ significantly in array counts (*p* = 0.734) or spacer counts (*p* = 0.414). Furthermore, prophage quality (completeness) showed no correlation with host CRISPR array counts (Spearman's *ρ* = 0.006, *p* = 0.874).

### Phylogenetic relationships of *S. mutans* phages and prophages

**Figure 5.**
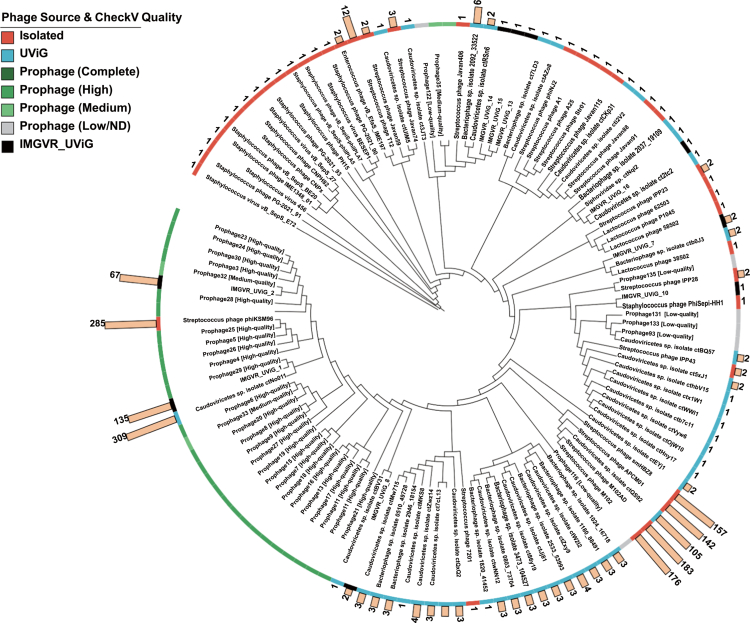
Circular tree of phage genomes targeted by *S. mutans* spacer matches and prophage. The outer strip indicates phage source and CheckV-based prophage quality, and the outer bar chart shows the log-transformed number of targeting *S. mutans* strains for each sequence.

To examine the phylogenetic relationships among the identified *S. mutans* (pro)phages, we constructed a maximum-likelihood tree based on terminase large subunit (TerL) proteins (Figure 5). In total, 93 TerL sequences were obtained from reference phages and UViGs (including 9 sequences extracted from the IMG/VR targets), and 36 putative TerL sequences were identified from the 186 predicted *S. mutans* prophages. The tree showed a non-uniform distribution of endogenous prophages. Most high-quality *S. mutans* prophages were concentrated in a major prophage-rich clade that also included *Streptococcus* phage phiKSM96, *Caudoviricetes* sp. ctNo011 and specific UViG (e.g. IMGVR_UViG_1 and IMGVR_UViG_2). In contrast, ctQS92 clustered with the previously isolated *S. mutans* phages smHBZ8, APCM01, M102, and M102AD in a separate clade with limited prophage representation. None of the environmental UViGs showed close phylogenetic proximity to this isolated phage clade. The remaining UViGs and several medium- and low-quality prophages were interspersed across other mixed branches rather than forming isolated outgroups.

To further characterize the genomic features defining these phylogenetic groups, we compared the functional modules of genomes across the two major clades based on existing VOG and Pfam-A annotations. The major prophage-rich clade (housing phiKSM96, ctNo011, IMGVR_UViG_1, and multiple predicted prophages) consistently shares conserved structural cassettes typical of temperate *Caudoviricetes*—such as homologous major capsid and portal proteins—alongside distinct site-specific integrase domains essential for the lysogenic cycle. In stark contrast, we did not detect homologous matches for these specific head structural VOGs within the isolated phage lineage (comprising ctQS92, M102, M102AD, APCM01, and smHBZ8).

## Discussion

In recent years, numerous studies have attempted to isolate bacteriophages targeting *S. mutans* from the oral cavity, informing future experimental and clincal work [16]. However, due to the narrow host range of bacteriophages and the ability of bacteria to evolve escape mechanisms [54], the isolation of *S. mutans* phages has proven extremely challenging. To date, only 5 phages have been successfully isolated. Yet, the true diversity of bacteriophages capable of infecting *S. mutans* in natural environments remains largely unknown. In this study, we utilized spacer sequences from the CRISPR systems in bacteria to predict potential *S. mutans* phages. Identifying phages via CRISPR spacers cannot definitively establish whether a host is currently sensitive or resistant to a specific virus. However, spacer acquisition proves that the phage can successfully recognize the host and inject its DNA, thereby triggering an adaptive immune response. This provides an indelible molecular record of historical phage-host encounters, even if a productive infection was aborted. This CRISPR-based approach, combined with systematic prophage mining, has proven to be a highly effective strategy for illuminating 'viral dark matter' and resolving complex virus-host interaction networks across diverse microbial ecosystems [55,56]. Using publicly available genomic sequences, we constructed the largest dataset of *S. mutans* genomes to date, incorporating the highest number of strains (Figure 1A & Table S1). Our analysis revealed that approximately 548 strains contained CRISPR systems. Regarding CRISPR typing, our results showed that type II-A was the most prevalent, followed by types I-C and I-E (Table S2). This finding differs slightly from previous studies, and we attribute this discrepancy to the larger scale of genomic data used in our analysis [57].

A previous study employed the same strategy and extracted ~8000 spacers (only 23.7% of spacers mapped to known targets) to analyze the impact of the CRISPR-Cas system on the horizontal gene transfer (HGT) and genomic evolution of *S. mutans* [21]. In contrast to this study, our study leverages these spacer repertoires to explicitly define the *S. mutans* 'viral dark matter' and prophage landscape. We obtained a total of 14,263 spacer sequences from these CRISPR systems (Figure 2A). We matched these spacers against all available gene databases and observed that only approximately 26% of the spacers could be matched, leaving the origin of the majority of spacers still unknown (Figure 2A). Among the known spacer sequences, most originated from exogenous bacteriophages, including 41 cultured (isolate-derived) phage genomes available in public databases (many of which were originally isolated from non-*S. mutans* hosts), and 51 metagenome-assembled viral genomes (UViGs) (Table S3). Among these, five correspond to already-isolated *S. mutans* phages, with at least 140 strains harboring spacers targeting them.

phiKSM96, a temperate phage isolated in recent years, has been previously reported to exhibit a broader host range compared to M102 and M102AD [18]. This study also found that more strains carry spacers targeting phiKSM96, confirming its wider host range. More importantly, our results suggest that *Caudoviricetes sp*. ctNo011 and ctQS92 may be potential temperate phages, with ctNo011 possibly having an even broader host range than phiKSM96. We also observed that *S. mutans* carries spacers targeting phages derived from other bacterial species, such as phage T12 from *Streptococcus pyogenes* and *Streptococcus* phage Javan74 from *Streptococcus pneumoniae* (Table S3). These findings raise the possibility that some phages originally described in other *Streptococcus* species could also interact with, and potentially infect, *S. mutans*. This interpretation is consistent with recent metaHiC-based evidence indicating that multi-host phage-bacterium associations are relatively common across ecosystems, challenging the traditional view of strictly narrow phage host ranges [58]. However, cross-species spacer matches should not be taken as direct evidence of cross-species infection. Alternative explanations include conserved sequences among streptococcal phages and horizontal gene transfer events within the highly plastic *S. mutans* genome [59]. *S. mutans* is a naturally competent species capable of taking up extracellular DNA from the environment, which serves as a well-recognized avenue for HGT [60]. It is possible that phages from other species were recorded by the CRISPR system during horizontal gene transfer events in the form of prophages [61,62]. The fact that many spacers identified in this study matched bacterial genomes further supports this hypothesis, as spacer hits in bacterial chromosomes are often enriched in prophage or other mobilome regions rather than in free phage genomes; a similar prophage-biased targeting pattern has been reported in natural *E. coli* isolates [63].

To briefly assess host genomic features associated with historical phage exposure, we performed a comparative genomic analysis of spacer-positive strains (Table S4). In phiKSM96 spacer-positive strains, transposase-related genes (TniB and Tn7) were enriched, suggesting increased genomic plasticity, which may favor foreign DNA acquisition and potentially facilitate phiKSM96 integration. We also detected YadA-like domains and SmpA/OmlA homologs. Although classical YadA systems are mainly described in Gram-negative bacteria, structurally related domains are widely found in Gram-positive surface adhesins [64], raising the possibility that these proteins contribute to cell-surface structures involved in phiKSM96 recognition. In smHBZ8 spacer-positive strains, specific RAMPs and Cas6 proteins were identified, consistent with a possible role in spacer acquisition. Notably, Glyco_transf_21 was uniquely enriched in this group. Given that rhamnose-glucose polysaccharides are key determinants of adsorption for several *S. mutans* phages [65–67], this glycosyltransferase may influence the surface polysaccharide architecture relevant to smHBZ8 binding. While these genomic associations provide insights into host-phage interactions, further experimental studies are needed to definitively characterize the molecular mechanisms modulating phage infection in *S. mutans*.

Prophages are the integrated form of temperate bacteriophages within the host genome. Since these sequences often carry virulence proteins, they are of significant importance to the pathogenicity of bacteria. In this study, we identified 186 prophage sequences from 735 *S. mutans* strains (Table S5). However, only 9 prophages had been reported in previous studies [68]. And the sequences previously labeled as ‘intact’ were more often assessed as medium- or high-quality prophage fragments in our analysis, rather than demonstrably complete phage genomes. To validate the reliability of our results, we reanalyzed the same dataset used in the previous study with both PHASTER [69] and VIBRANT [39]. The two tools produced markedly different results: the PHASTER-based workflow reproduced the earlier findings, whereas VIBRANT yielded results consistent with those presented here. We attribute this discrepancy primarily to differences in prediction strategy between the two programs. Our choice of VIBRANT was supported by independent benchmarking studies showing that it performs well overall in prophage prediction [70]. By scaling up the analysis to 735 genomes, we identified 186 prophage regions (Table S5), expanding the currently recognized prophage repertoire of *S. mutans*. Notably, we detected a high-quality region matching the *Streptococcus* satellite phage Javan66 (Figure 4C). Despite the prevalence of satellite prophages across the genus *Streptococcus* [67], they remain poorly characterized in *S. mutans*. This finding expands the known *S. mutans* mobilome by incorporating satellite phage-related elements into its repertoire. Moreover, the high proportion of ‘low-quality’ prophages (59.1%) suggests *S. mutans* predominantly harbors ‘decaying’ remnants. Lacking the essential structural genes required for a complete viral life cycle, these low-quality elements are highly unlikely to be induced like the intact prophage phiKSM96, thus limiting their direct clinical potential. Instead, they more likely serve as a genetic reservoir for the host bacterium, potentially contributing adaptive traits such as virulence factors or defense systems.

Our statistical analyses further clarified the relationships among CRISPR-Cas features of *S. mutans*. CRISPR array counts, spacer counts, and targeted phage counts were positively correlated, indicating that strains with larger spacer repertoires tend to target a broader range of phages. Strains carrying multiple CRISPR-Cas subtypes also showed higher array counts and broader phage-targeting profiles than strains with only one subtype, suggesting enhanced defense capacity. In contrast, these CRISPR-Cas features were not associated with prophage presence or prophage quality. This suggests that integrated prophages may not be strongly constrained by the current CRISPR-Cas status of the host, possibly because they were acquired before spacer acquisition or escaped recognition after integration.

Integrating IMG/VR UViGs further emphasized that cultured *S. mutans* phages represent only a small fraction of the viral diversity that can interact with this host. The pronounced phylogenetic divergence among UViGs, integrated prophage elements, and known isolates supports the presence of extensive uncultured viral lineages in oral microbiomes. Protein-level phylogenetic analysis provided additional context for the major spacer-targeted phages. In the TerL tree, IMGVR_UViG_1 and ctNo011 grouped with phiKSM96 and multiple *S. mutans* prophages, whereas ctQS92 clustered with previously isolated *S. mutans* phages, including smHBZ8, APCM01, M102, and M102AD. This structural dichotomy elegantly explains the current isolation bias toward the latter clade, strengthening our confidence in culturing ctQS92. Furthermore, the recent isolation of phiKSM96 demonstrates that isolating elusive 'dark matter' candidates remains entirely feasible under optimized conditions.

In summary, by integrating spacer-based inference and prophage mining, we delineated a historical interaction landscape between *S. mutans* and diverse viral lineages. We also generated a genomic catalog of candidate phages and prophage regions. For prevalent candidates like ctNo011, IMGVR_UViG_1, and ctQS92, this catalog details their genomic coordinates, CheckV quality estimates, functional cargo annotations (e.g. CAZy, PADLOC), and specific host-strain mappings. In the future, specific bacteriophages could be isolated by constructing specific host strains or host strains with immune system deficiencies. For example, previous research has successfully isolated the temperate bacteriophage phiKSM96 based on prophage identification [18]. Our findings reveal that the bacteriophage ctNo011 can be mapped to 309 host strains. Therefore, we speculate that, based on our data, ctNo011 represents a highly prevalent viral lineage that should be prioritized for targeted isolation and in vitro host-range characterization. Furthermore, although phage isolation remains challenging, recent advances in engineered phage research have employed methods such as combining receptor-binding proteins (RBPs) with nanomaterials or drugs to achieve targeted antibacterial effects [71].

The limitations of this study are as follows. First, most currently available genomic data on phages and bacterial strains are derived from Next-generation sequencing (NGS). Due to read length limitation, NGS struggles to accurately resolve repetitive sequences. Consequently, during analysis, many spacer sequences within CRISPR arrays or phage sequences assembled from metagenomic data may be missing, leading to reduced accuracy. Second, the limited availability of known phage genomic data means that approximately 75% of the spacer sequences identified in this study could not be matched to known DNA sequences via BLAST, which may introduce a degree of bias into our results. In recent years, the long-read sequencing technologies have demonstrated significant potential in resolving repetitive sequences and phage genomes [72]. Future exploration based on long-read sequencing data is expected to yield more comprehensive information on *S. mutans* bacteriophages.

## Supplementary Material

Table_S5.xlsx Table_S5.xlsx

Supplementary.docx Supplementary.docx

## Data Availability

All data analyzed in this study are publicly available. *Streptococcus mutans* genome assemblies were obtained from the Human Oral Microbiome Database (HOMD) and NCBI GenBank/RefSeq, as well as previously published datasets cited in the Methods. Accession numbers for all genomes included in the analysis are provided in Supplementary Table S1.
