# Supplementary material for: CRISPR spacer profiling and prophage mining reveal diverse bacteriophages associated with Streptococcus Mutans
Source: J Oral Microbiol. 2026 May 21;18(1):2674332. doi: 10.1080/20002297.2026.2674332 (PMC13195714; doi:10.1080/20002297.2026.2674332)
Supplement: Supplementary.docx [file ZJOM_A_2674332_SM0290.docx]

**Table S1. Metadata and data sources of *Streptococcus mutans* genomes included in this study.**

| smu | strain_id | country | data source | |
| --- | --- | --- | --- | --- |
| smu1 | GCA_001558215.1 | Papua New Guinea | | Walker AR |
| smu2 | GCA_001069835.1 | USA | | Walker AR |
| smu3 | GCA_001068415.1 | USA | | Walker AR |
| smu4 | GCA_001073145.1 | USA | | Walker AR |
| smu5 | GCA_001703615.1 | Italy | | Walker AR |
| smu6 | GCA_001625005.1 | USA | | Walker AR |
| smu7 | GCA_000339695.1 | Brazil | | Walker AR |
| smu8 | GCA_000339715.1 | Brazil | | Walker AR |
| smu9 | GCA_000339255.1 | Brazil | | Walker AR |
| smu10 | GCA_000339515.1 | Iceland | | Walker AR |
| smu11 | GCA_000339155.1 | USA | | Walker AR |
| smu12 | GCA_000339235.1 | Brazil | | Walker AR |
| smu13 | GCA_000340095.1 | Brazil | | Walker AR |
| smu14 | GCA_000339655.1 | USA | | Walker AR |
| smu15 | GCA_000339535.1 | Iceland | | Walker AR |
| smu16 | GCA_000340155.1 | Iceland | | Walker AR |
| smu17 | GCA_000339195.1 | Brazil | | Walker AR |
| smu18 | GCA_000339735.1 | Brazil | | Walker AR |
| smu19 | GCA_000339675.1 | Brazil | | Walker AR |
| smu20 | GCA_000339175.1 | Brazil | | Walker AR |
| smu21 | GCA_000339215.1 | Brazil | | Walker AR |
| smu22 | GCA_000347795.1 | United Kingdom | | Walker AR |
| smu23 | GCA_000339275.1 | Brazil | | Walker AR |
| smu24 | GCA_000340115.1 | Iceland | | Walker AR |
| smu25 | GCA_000339595.1 | Brazil | | Walker AR |
| smu26 | GCA_000339775.1 | United Kingdom | | Walker AR |
| smu27 | GCA_000339315.1 | United Kingdom | | Walker AR |
| smu28 | GCA_000347855.1 | Germany | | Walker AR |
| smu29 | GCA_000347875.1 | Germany | | Walker AR |
| smu30 | GCA_000339555.1 | Iceland | | Walker AR |
| smu31 | unknown | unknown | | Walker AR |
| smu32 | GCA_000522565.1 | USA | | Walker AR |
| smu33 | unknown | unknown | | Walker AR |
| smu34 | unknown | unknown | | Walker AR |
| smu35 | unknown | unknown | | Walker AR |
| smu36 | GCA_000522625.1 | USA | | Walker AR |
| smu37 | unknown | unknown | | Walker AR |
| smu38 | GCA_000522725.1 | USA | | Walker AR |
| smu39 | unknown | unknown | | Walker AR |
| smu40 | unknown | unknown | | Walker AR |
| smu41 | unknown | unknown | | Walker AR |
| smu42 | unknown | unknown | | Walker AR |
| smu43 | unknown | unknown | | Walker AR |
| smu44 | GCA_000522605.1 | USA | | Walker AR |
| smu45 | GCA_000522585.1 | USA | | Walker AR |
| smu46 | GCA_000522885.1 | USA | | Walker AR |
| smu47 | unknown | unknown | | Walker AR |
| smu48 | unknown | unknown | | Walker AR |
| smu49 | GCA_000522845.1 | USA | | Walker AR |
| smu50 | GCA_000375505.1 | USA | | Walker AR |
| smu51 | GCA_000339815.1 | United Kingdom | | Walker AR |
| smu52 | GCA_000271865.1 | USA | | Walker AR |
| smu53 | GCA_000347815.1 | Germany | | Walker AR |
| smu54 | GCA_000347835.1 | Germany | | Walker AR |
| smu55 | GCA_000284575.1 | Japan | | Walker AR |
| smu56 | GCA_000339835.1 | United Kingdom | | Walker AR |
| smu57 | GCA_000339135.1 | Brazil | | Walker AR |
| smu58 | GCA_000339935.1 | United Kingdom | | Walker AR |
| smu59 | GCA_000339335.1 | United Kingdom | | Walker AR |
| smu60 | GCA_000339955.1 | United Kingdom | | Walker AR |
| smu61 | GCA_000339855.1 | United Kingdom | | Walker AR |
| smu62 | GCA_000339975.1 | United Kingdom | | Walker AR |
| smu63 | GCA_000347895.1 | Denmark | | Walker AR |
| smu64 | GCA_000339395.1 | United Kingdom | | Walker AR |
| smu65 | unknown | unknown | | Walker AR |
| smu66 | GCA_000340075.1 | United Kingdom | | Walker AR |
| smu67 | GCA_000339875.1 | United Kingdom | | Walker AR |
| smu68 | GCA_000339895.1 | United Kingdom | | Walker AR |
| smu69 | GCA_000339495.1 | United Kingdom | | Walker AR |
| smu70 | GCA_000339915.1 | United Kingdom | | Walker AR |
| smu71 | unknown | unknown | | Walker AR |
| smu72 | GCA_000091645.1 | Japan | | Walker AR |
| smu73 | GCA_000339415.1 | USA | | Walker AR |
| smu74 | GCA_000339755.1 | USA | | Walker AR |
| smu75 | GCA_000339635.1 | USA | | Walker AR |
| smu76 | GCA_000496535.1 | China | | Walker AR |
| smu77 | GCA_000496555.1 | China | | Walker AR |
| smu78 | GCA_000339115.1 | Brazil | | Walker AR |
| smu79 | GCA_000339055.1 | Turkey | | Walker AR |
| smu80 | GCA_000339615.1 | South Africa | | Walker AR |
| smu81 | GCA_000339075.1 | South Africa | | Walker AR |
| smu82 | GCA_000340015.1 | USA | | Walker AR |
| smu83 | unknown | unknown | | Walker AR |
| smu84 | GCA_000339435.1 | USA | | Walker AR |
| smu85 | GCA_000339575.1 | China | | Walker AR |
| smu86 | GCA_000340135.1 | China | | Walker AR |
| smu87 | GCA_000339455.1 | China | | Walker AR |
| smu88 | GCA_000340035.1 | United Kingdom | | Walker AR |
| smu89 | GCA_000340055.1 | United Kingdom | | Walker AR |
| smu90 | unknown | unknown | | Walker AR |
| smu91 | GCA_000339375.1 | United Kingdom | | Walker AR |
| smu92 | GCA_000228745.2 | Brazil | | Walker AR |
| smu93 | unknown | unknown | | Walker AR |
| smu94 | GCA_000228785.2 | Brazil | | Walker AR |
| smu95 | unknown | unknown | | Walker AR |
| smu96 | GCA_000228825.2 | Brazil | | Walker AR |
| smu97 | GCA_000228845.2 | Brazil | | Walker AR |
| smu98 | GCA_000228865.2 | Brazil | | Walker AR |
| smu99 | GCA_000228885.2 | Brazil | | Walker AR |
| smu100 | GCA_000228905.2 | Brazil | | Walker AR |
| smu101 | GCA_000228925.2 | Brazil | | Walker AR |
| smu102 | GCA_000228945.2 | Brazil | | Walker AR |
| smu103 | GCA_000228965.2 | Brazil | | Walker AR |
| smu104 | GCA_000228985.2 | Brazil | | Walker AR |
| smu105 | GCA_000229005.2 | Brazil | | Walker AR |
| smu106 | GCA_000229025.2 | Brazil | | Walker AR |
| smu107 | GCA_000229045.2 | Brazil | | Walker AR |
| smu108 | GCA_000229065.2 | Brazil | | Walker AR |
| smu109 | GCA_000229085.2 | Brazil | | Walker AR |
| smu110 | GCA_000229105.2 | Brazil | | Walker AR |
| smu111 | GCA_000229125.2 | Brazil | | Walker AR |
| smu112 | GCA_000229145.2 | Brazil | | Walker AR |
| smu113 | GCA_000229165.2 | Brazil | | Walker AR |
| smu114 | GCA_000229185.2 | Brazil | | Walker AR |
| smu115 | GCA_000229205.2 | Brazil | | Walker AR |
| smu116 | unknown | unknown | | Walker AR |
| smu117 | GCA_000229245.2 | Brazil | | Walker AR |
| smu118 | GCA_000229265.2 | Brazil | | Walker AR |
| smu119 | GCA_000229285.2 | Brazil | | Walker AR |
| smu120 | GCA_000229305.2 | Brazil | | Walker AR |
| smu121 | GCA_000229325.2 | Brazil | | Walker AR |
| smu122 | unknown | unknown | | Walker AR |
| smu123 | GCA_000229365.2 | Brazil | | Walker AR |
| smu124 | GCA_000229385.2 | Brazil | | Walker AR |
| smu125 | GCA_000229405.2 | Brazil | | Walker AR |
| smu126 | GCA_000229425.2 | Brazil | | Walker AR |
| smu127 | GCA_000229445.2 | Brazil | | Walker AR |
| smu128 | GCA_000229465.2 | Brazil | | Walker AR |
| smu129 | GCA_000229485.2 | Brazil | | Walker AR |
| smu130 | GCA_000229505.2 | Brazil | | Walker AR |
| smu131 | GCA_000229525.2 | Brazil | | Walker AR |
| smu132 | unknown | unknown | | Walker AR |
| smu133 | GCA_000229565.2 | Brazil | | Walker AR |
| smu134 | GCA_000229585.2 | Brazil | | Walker AR |
| smu135 | GCA_000229605.2 | Brazil | | Walker AR |
| smu136 | GCA_000229625.2 | Brazil | | Walker AR |
| smu137 | GCA_000229645.2 | Brazil | | Walker AR |
| smu138 | GCA_000229665.2 | Brazil | | Walker AR |
| smu139 | GCA_000229685.2 | Brazil | | Walker AR |
| smu140 | GCA_000229705.2 | Brazil | | Walker AR |
| smu141 | GCA_000229725.2 | Brazil | | Walker AR |
| smu142 | GCA_000229745.2 | Brazil | | Walker AR |
| smu143 | GCA_000229765.2 | Brazil | | Walker AR |
| smu144 | GCA_000229785.2 | Brazil | | Walker AR |
| smu145 | GCA_000229805.2 | Brazil | | Walker AR |
| smu146 | GCA_000229825.2 | Brazil | | Walker AR |
| smu147 | GCA_000229845.2 | Brazil | | Walker AR |
| smu148 | GCA_000229865.2 | Brazil | | Walker AR |
| smu149 | GCA_000229885.2 | Brazil | | Walker AR |
| smu150 | GCA_000229905.2 | Brazil | | Walker AR |
| smu151 | GCA_000229925.2 | Brazil | | Walker AR |
| smu152 | GCA_000229945.2 | Brazil | | Walker AR |
| smu153 | GCA_000229965.2 | Brazil | | Walker AR |
| smu154 | GCA_000229985.2 | Brazil | | Walker AR |
| smu155 | GCA_000230005.2 | Brazil | | Walker AR |
| smu156 | GCA_000230025.2 | Brazil | | Walker AR |
| smu157 | GCA_000230045.2 | Brazil | | Walker AR |
| smu158 | GCA_000230065.2 | Brazil | | Walker AR |
| smu159 | GCA_000230085.2 | Brazil | | Walker AR |
| smu160 | GCA_000230105.2 | Brazil | | Walker AR |
| smu161 | GCA_000230125.2 | Brazil | | Walker AR |
| smu162 | GCA_000230145.2 | Brazil | | Walker AR |
| smu163 | GCA_000230165.2 | Brazil | | Walker AR |
| smu164 | GCA_000230185.2 | Brazil | | Walker AR |
| smu165 | GCA_000230205.2 | Brazil | | Walker AR |
| smu166 | GCA_000230225.2 | Brazil | | Walker AR |
| smu167 | GCA_000339795.1 | United Kingdom | | Walker AR |
| smu168 | GCA_000339475.1 | Turkey | | Walker AR |
| smu169 | GCA_000340175.1 | Turkey | | Walker AR |
| smu170 | GCA_000007465.2 | USA | | Walker AR |
| smu171 | GCA_000817065.1 | China | | Walker AR |
| smu172 | GCA_000339995.1 | United Kingdom | | Walker AR |
| smu173 | unknown | unknown | | Walker AR |
| smu174 | unknown | unknown | | Walker AR |
| smu175 | unknown | unknown | | Walker AR |
| smu176 | unknown | unknown | | Walker AR |
| smu177 | unknown | unknown | | Walker AR |
| smu178 | unknown | unknown | | Walker AR |
| smu179 | unknown | unknown | | Walker AR |
| smu180 | unknown | unknown | | Walker AR |
| smu181 | unknown | unknown | | Walker AR |
| smu182 | unknown | unknown | | Walker AR |
| smu183 | unknown | unknown | | Walker AR |
| smu184 | unknown | unknown | | Walker AR |
| smu185 | unknown | unknown | | Walker AR |
| smu186 | unknown | unknown | | Walker AR |
| smu187 | unknown | unknown | | Walker AR |
| smu188 | unknown | unknown | | Walker AR |
| smu189 | unknown | unknown | | Walker AR |
| smu190 | unknown | unknown | | Walker AR |
| smu191 | unknown | unknown | | Walker AR |
| smu192 | unknown | unknown | | Walker AR |
| smu193 | unknown | unknown | | Walker AR |
| smu194 | unknown | unknown | | Walker AR |
| smu195 | unknown | unknown | | Walker AR |
| smu196 | unknown | unknown | | Walker AR |
| smu197 | unknown | unknown | | Walker AR |
| smu198 | unknown | unknown | | Walker AR |
| smu199 | unknown | unknown | | Walker AR |
| smu200 | unknown | unknown | | Walker AR |
| smu201 | unknown | unknown | | Walker AR |
| smu202 | unknown | unknown | | Walker AR |
| smu203 | unknown | unknown | | Walker AR |
| smu204 | unknown | unknown | | Walker AR |
| smu205 | unknown | unknown | | Walker AR |
| smu206 | unknown | unknown | | Walker AR |
| smu207 | unknown | unknown | | Walker AR |
| smu208 | unknown | unknown | | Walker AR |
| smu209 | unknown | unknown | | Walker AR |
| smu210 | unknown | unknown | | Walker AR |
| smu211 | unknown | unknown | | Walker AR |
| smu212 | unknown | unknown | | Walker AR |
| smu213 | unknown | unknown | | Walker AR |
| smu214 | unknown | unknown | | Walker AR |
| smu215 | unknown | unknown | | Walker AR |
| smu216 | unknown | unknown | | Walker AR |
| smu217 | unknown | unknown | | Walker AR |
| smu218 | unknown | unknown | | Walker AR |
| smu219 | unknown | unknown | | Walker AR |
| smu220 | unknown | unknown | | Walker AR |
| smu221 | unknown | unknown | | Walker AR |
| smu222 | unknown | unknown | | Walker AR |
| smu223 | unknown | unknown | | Walker AR |
| smu224 | unknown | unknown | | Walker AR |
| smu225 | unknown | unknown | | Walker AR |
| smu226 | unknown | unknown | | Walker AR |
| smu227 | unknown | unknown | | Walker AR |
| smu228 | unknown | unknown | | Walker AR |
| smu229 | unknown | unknown | | Walker AR |
| smu230 | unknown | unknown | | Walker AR |
| smu231 | unknown | unknown | | Walker AR |
| smu232 | unknown | unknown | | Walker AR |
| smu233 | unknown | unknown | | Walker AR |
| smu234 | unknown | unknown | | Walker AR |
| smu235 | unknown | unknown | | Walker AR |
| smu236 | unknown | unknown | | Walker AR |
| smu237 | unknown | unknown | | Walker AR |
| smu238 | unknown | unknown | | Walker AR |
| smu239 | unknown | unknown | | Walker AR |
| smu240 | unknown | unknown | | Walker AR |
| smu241 | unknown | unknown | | Walker AR |
| smu242 | unknown | unknown | | Walker AR |
| smu243 | unknown | unknown | | Walker AR |
| smu244 | unknown | unknown | | Walker AR |
| smu245 | unknown | unknown | | Walker AR |
| smu246 | unknown | unknown | | Walker AR |
| smu247 | unknown | unknown | | Walker AR |
| smu248 | unknown | unknown | | Walker AR |
| smu249 | unknown | unknown | | Walker AR |
| smu250 | unknown | unknown | | Walker AR |
| smu251 | unknown | unknown | | Walker AR |
| smu252 | unknown | unknown | | Walker AR |
| smu253 | unknown | unknown | | Walker AR |
| smu254 | unknown | unknown | | Walker AR |
| smu255 | unknown | unknown | | Walker AR |
| smu256 | unknown | unknown | | Walker AR |
| smu257 | unknown | unknown | | Walker AR |
| smu258 | unknown | unknown | | Walker AR |
| smu259 | unknown | unknown | | Walker AR |
| smu260 | unknown | unknown | | Walker AR |
| smu261 | unknown | unknown | | Walker AR |
| smu262 | unknown | unknown | | Walker AR |
| smu263 | unknown | unknown | | Walker AR |
| smu264 | unknown | unknown | | Walker AR |
| smu265 | unknown | unknown | | Walker AR |
| smu266 | unknown | unknown | | Walker AR |
| smu267 | unknown | unknown | | Walker AR |
| smu268 | unknown | unknown | | Walker AR |
| smu269 | unknown | unknown | | Walker AR |
| smu270 | unknown | unknown | | Walker AR |
| smu271 | unknown | unknown | | Walker AR |
| smu272 | unknown | unknown | | Walker AR |
| smu273 | unknown | unknown | | Walker AR |
| smu274 | unknown | unknown | | Walker AR |
| smu275 | unknown | unknown | | Walker AR |
| smu276 | unknown | unknown | | Walker AR |
| smu277 | unknown | unknown | | Walker AR |
| smu278 | unknown | unknown | | Walker AR |
| smu279 | unknown | unknown | | Walker AR |
| smu280 | unknown | unknown | | Walker AR |
| smu281 | unknown | unknown | | Walker AR |
| smu282 | unknown | unknown | | Walker AR |
| smu283 | unknown | unknown | | Walker AR |
| smu284 | unknown | unknown | | Walker AR |
| smu285 | unknown | unknown | | Walker AR |
| smu286 | unknown | unknown | | Walker AR |
| smu287 | unknown | unknown | | Walker AR |
| smu288 | unknown | unknown | | Walker AR |
| smu289 | unknown | unknown | | Walker AR |
| smu290 | unknown | unknown | | Walker AR |
| smu291 | unknown | unknown | | Walker AR |
| smu292 | unknown | unknown | | Walker AR |
| smu293 | unknown | unknown | | Walker AR |
| smu294 | unknown | unknown | | Walker AR |
| smu295 | unknown | unknown | | Walker AR |
| smu296 | unknown | unknown | | Walker AR |
| smu297 | unknown | unknown | | Walker AR |
| smu298 | unknown | unknown | | Walker AR |
| smu299 | unknown | unknown | | Walker AR |
| smu300 | unknown | unknown | | Walker AR |
| smu301 | unknown | unknown | | Walker AR |
| smu302 | unknown | unknown | | Walker AR |
| smu303 | unknown | unknown | | Walker AR |
| smu304 | unknown | unknown | | Walker AR |
| smu305 | unknown | unknown | | Walker AR |
| smu306 | unknown | unknown | | Walker AR |
| smu307 | unknown | unknown | | Walker AR |
| smu308 | unknown | unknown | | Walker AR |
| smu309 | unknown | unknown | | Walker AR |
| smu310 | unknown | unknown | | Walker AR |
| smu311 | unknown | unknown | | Walker AR |
| smu312 | unknown | unknown | | Walker AR |
| smu313 | unknown | unknown | | Walker AR |
| smu314 | unknown | unknown | | Walker AR |
| smu315 | unknown | unknown | | Walker AR |
| smu316 | unknown | unknown | | Walker AR |
| smu317 | unknown | unknown | | Walker AR |
| smu318 | unknown | unknown | | Walker AR |
| smu319 | unknown | unknown | | Walker AR |
| smu320 | unknown | unknown | | Walker AR |
| smu321 | unknown | unknown | | Walker AR |
| smu322 | unknown | unknown | | Walker AR |
| smu323 | unknown | unknown | | Walker AR |
| smu324 | unknown | unknown | | Walker AR |
| smu325 | unknown | unknown | | Walker AR |
| smu326 | unknown | unknown | | Walker AR |
| smu327 | unknown | unknown | | Walker AR |
| smu328 | unknown | unknown | | Walker AR |
| smu329 | unknown | unknown | | Walker AR |
| smu330 | unknown | unknown | | Walker AR |
| smu331 | unknown | unknown | | Walker AR |
| smu332 | unknown | unknown | | Walker AR |
| smu333 | unknown | unknown | | Walker AR |
| smu334 | unknown | unknown | | Walker AR |
| smu335 | unknown | unknown | | Walker AR |
| smu336 | unknown | unknown | | Walker AR |
| smu337 | unknown | unknown | | Walker AR |
| smu338 | unknown | unknown | | Walker AR |
| smu339 | unknown | unknown | | Walker AR |
| smu340 | unknown | unknown | | Walker AR |
| smu341 | unknown | unknown | | Walker AR |
| smu342 | unknown | unknown | | Walker AR |
| smu343 | unknown | unknown | | Walker AR |
| smu344 | unknown | unknown | | Walker AR |
| smu345 | unknown | unknown | | Walker AR |
| smu346 | unknown | unknown | | Walker AR |
| smu347 | unknown | unknown | | Walker AR |
| smu348 | unknown | unknown | | Walker AR |
| smu349 | unknown | unknown | | Walker AR |
| smu350 | unknown | unknown | | Walker AR |
| smu351 | unknown | unknown | | Walker AR |
| smu352 | unknown | unknown | | Walker AR |
| smu353 | unknown | unknown | | Walker AR |
| smu354 | unknown | unknown | | Walker AR |
| smu355 | unknown | unknown | | Walker AR |
| smu356 | unknown | unknown | | Walker AR |
| smu357 | unknown | unknown | | Walker AR |
| smu358 | unknown | unknown | | Walker AR |
| smu359 | unknown | unknown | | Walker AR |
| smu360 | unknown | unknown | | Walker AR |
| smu361 | unknown | unknown | | Walker AR |
| smu362 | unknown | unknown | | Walker AR |
| smu363 | unknown | unknown | | Walker AR |
| smu364 | unknown | unknown | | Walker AR |
| smu365 | unknown | unknown | | Walker AR |
| smu366 | unknown | unknown | | Walker AR |
| smu367 | unknown | unknown | | Walker AR |
| smu368 | unknown | unknown | | Walker AR |
| smu369 | unknown | unknown | | Walker AR |
| smu370 | unknown | unknown | | Walker AR |
| smu371 | unknown | unknown | | Walker AR |
| smu372 | unknown | unknown | | Walker AR |
| smu373 | unknown | unknown | | Walker AR |
| smu374 | unknown | unknown | | Walker AR |
| smu375 | unknown | unknown | | Walker AR |
| smu376 | unknown | unknown | | Walker AR |
| smu377 | unknown | unknown | | Walker AR |
| smu378 | unknown | unknown | | Walker AR |
| smu379 | unknown | unknown | | Walker AR |
| smu380 | unknown | unknown | | Walker AR |
| smu381 | unknown | unknown | | Walker AR |
| smu382 | unknown | unknown | | Walker AR |
| smu383 | unknown | unknown | | Walker AR |
| smu384 | unknown | unknown | | Walker AR |
| smu385 | unknown | unknown | | Walker AR |
| smu386 | unknown | unknown | | Walker AR |
| smu387 | unknown | unknown | | Walker AR |
| smu388 | unknown | unknown | | Walker AR |
| smu389 | unknown | unknown | | Walker AR |
| smu390 | unknown | unknown | | Walker AR |
| smu391 | unknown | unknown | | Walker AR |
| smu392 | unknown | unknown | | Walker AR |
| smu393 | unknown | unknown | | Walker AR |
| smu394 | unknown | unknown | | Walker AR |
| smu395 | unknown | unknown | | Walker AR |
| smu396 | unknown | unknown | | Walker AR |
| smu397 | unknown | unknown | | Walker AR |
| smu398 | unknown | unknown | | Walker AR |
| smu399 | unknown | unknown | | Walker AR |
| smu400 | unknown | unknown | | Walker AR |
| smu401 | unknown | unknown | | Walker AR |
| smu402 | unknown | unknown | | Walker AR |
| smu403 | unknown | unknown | | Walker AR |
| smu404 | unknown | unknown | | Walker AR |
| smu405 | unknown | unknown | | Walker AR |
| smu406 | unknown | unknown | | Walker AR |
| smu407 | unknown | unknown | | Walker AR |
| smu408 | unknown | unknown | | Walker AR |
| smu409 | unknown | unknown | | Walker AR |
| smu410 | unknown | unknown | | Walker AR |
| smu411 | unknown | unknown | | Walker AR |
| smu412 | unknown | unknown | | Walker AR |
| smu413 | unknown | unknown | | Walker AR |
| smu414 | unknown | unknown | | Walker AR |
| smu415 | unknown | unknown | | Walker AR |
| smu416 | unknown | unknown | | Walker AR |
| smu417 | unknown | unknown | | Walker AR |
| smu418 | unknown | unknown | | Walker AR |
| smu419 | unknown | unknown | | Walker AR |
| smu420 | unknown | unknown | | Walker AR |
| smu421 | unknown | unknown | | Walker AR |
| smu422 | unknown | unknown | | Walker AR |
| smu423 | unknown | unknown | | Walker AR |
| smu424 | unknown | unknown | | Walker AR |
| smu425 | unknown | unknown | | Walker AR |
| smu426 | unknown | unknown | | Walker AR |
| smu427 | unknown | unknown | | Walker AR |
| smu428 | unknown | unknown | | Walker AR |
| smu429 | unknown | unknown | | Walker AR |
| smu430 | unknown | unknown | | Walker AR |
| smu431 | unknown | unknown | | Walker AR |
| smu432 | unknown | unknown | | Walker AR |
| smu433 | unknown | unknown | | Walker AR |
| smu434 | unknown | unknown | | Walker AR |
| smu435 | unknown | unknown | | Walker AR |
| smu436 | unknown | unknown | | Walker AR |
| smu437 | unknown | unknown | | Walker AR |
| smu438 | unknown | unknown | | Walker AR |
| smu439 | unknown | unknown | | Walker AR |
| smu440 | unknown | unknown | | Walker AR |
| smu441 | unknown | unknown | | Walker AR |
| smu442 | unknown | unknown | | Walker AR |
| smu443 | unknown | unknown | | Walker AR |
| smu444 | unknown | unknown | | Walker AR |
| smu445 | unknown | unknown | | Walker AR |
| smu446 | unknown | unknown | | Walker AR |
| smu447 | unknown | unknown | | Walker AR |
| smu448 | unknown | unknown | | Walker AR |
| smu449 | unknown | unknown | | Walker AR |
| smu450 | unknown | unknown | | Walker AR |
| smu451 | unknown | unknown | | Walker AR |
| smu452 | unknown | unknown | | Walker AR |
| smu453 | unknown | unknown | | Walker AR |
| smu454 | unknown | unknown | | Walker AR |
| smu455 | unknown | unknown | | Walker AR |
| smu456 | unknown | unknown | | Walker AR |
| smu457 | unknown | unknown | | Walker AR |
| smu458 | unknown | unknown | | Walker AR |
| smu459 | unknown | unknown | | Walker AR |
| smu460 | unknown | unknown | | Walker AR |
| smu461 | unknown | unknown | | Walker AR |
| smu462 | unknown | unknown | | Walker AR |
| smu463 | unknown | unknown | | Walker AR |
| smu464 | unknown | unknown | | Walker AR |
| smu465 | unknown | unknown | | Walker AR |
| smu466 | unknown | unknown | | Walker AR |
| smu467 | unknown | unknown | | Walker AR |
| smu468 | unknown | unknown | | Walker AR |
| smu469 | unknown | unknown | | Walker AR |
| smu470 | unknown | unknown | | Walker AR |
| smu471 | unknown | unknown | | Walker AR |
| smu472 | unknown | unknown | | Walker AR |
| smu473 | unknown | unknown | | Walker AR |
| smu474 | unknown | unknown | | Walker AR |
| smu475 | unknown | unknown | | Walker AR |
| smu476 | unknown | unknown | | Walker AR |
| smu477 | GCA_014621675.1 | USA | | HOMD |
| smu478 | GCA_000522765.1 | USA | | HOMD |
| smu479 | GCA_000522805.2 | USA | | HOMD |
| smu480 | GCA_000522665.1 | USA | | HOMD |
| smu481 | GCA_000522785.1 | USA | | HOMD |
| smu482 | GCA_000522685.1 | USA | | HOMD |
| smu483 | GCA_000522645.1 | USA | | HOMD |
| smu484 | GCA_000522905.1 | USA | | HOMD |
| smu485 | GCA_000522745.1 | USA | | HOMD |
| smu486 | GCA_000522925.1 | USA | | HOMD |
| smu487 | GCA_000522945.1 | USA | | HOMD |
| smu488 | GCA_000522705.1 | USA | | HOMD |
| smu489 | GCA_000522825.1 | USA | | HOMD |
| smu490 | GCA_002157665.1 | Japan | | HOMD |
| smu491 | GCA_902365065.1 | United Kingdom | | HOMD |
| smu492 | GCA_901875565.1 | USA | | HOMD |
| smu493 | GCA_006739205.1 | Japan | | HOMD |
| smu494 | GCA_000339295.1 | United Kingdom | | HOMD |
| smu495 | GCA_000339355.1 | Japan | | HOMD |
| smu496 | GCA_000339095.1 | USA | | HOMD |
| smu497 | GCA_000522865.1 | USA | | HOMD |
| smu498 | GCA_015670115.1 | USA | | HOMD |
| smu499 | GCA_015668935.1 | USA | | HOMD |
| smu500 | GCA_015556125.1 | USA | | HOMD |
| smu501 | GCA_014842815.3 | USA | | HOMD |
| smu502 | GCA_006386535.1 | China | | HOMD |
| smu503 | GCA_003466855.1 | China | | HOMD |
| smu504 | GCA_002179995.1 | USA | | HOMD |
| smu505 | GCA_021013185.1 | China | | HOMD |
| smu506 | GCA_015670285.1 | USA | | HOMD |
| smu507 | GCA_015669655.1 | USA | | HOMD |
| smu508 | GCA_018619415.1 | USA | | HOMD |
| smu509 | GCA_019048645.1 | Germany | | HOMD |
| smu510 | GCA_002083175.2 | USA | | HOMD |
| smu511 | GCA_012273155.1 | USA | | HOMD |
| smu512 | GCA_002155285.1 | South Korea | | HOMD |
| smu513 | GCA_020531125.1 | Japan | | HOMD |
| smu514 | GCA_020529965.1 | Japan | | HOMD |
| smu515 | GCA_020529995.1 | Japan | | HOMD |
| smu516 | GCA_020529445.1 | Japan | | HOMD |
| smu517 | GCA_020529385.1 | Japan | | HOMD |
| smu518 | GCA_020529945.1 | Japan | | HOMD |
| smu519 | GCA_020529915.1 | Japan | | HOMD |
| smu520 | GCA_020529705.1 | Japan | | HOMD |
| smu521 | GCA_020529405.1 | Japan | | HOMD |
| smu522 | GCA_020529255.1 | Japan | | HOMD |
| smu523 | GCA_020529325.1 | Japan | | HOMD |
| smu524 | GCA_020529305.1 | Japan | | HOMD |
| smu525 | GCA_020529685.1 | Japan | | HOMD |
| smu526 | GCA_020530845.1 | Japan | | HOMD |
| smu527 | GCA_020531625.1 | Japan | | HOMD |
| smu528 | GCA_020529225.1 | Japan | | HOMD |
| smu529 | GCA_020529245.1 | Japan | | HOMD |
| smu530 | GCA_020531665.1 | Japan | | HOMD |
| smu531 | GCA_020530685.1 | Japan | | HOMD |
| smu532 | GCA_020530645.1 | Japan | | HOMD |
| smu533 | GCA_020531085.1 | Japan | | HOMD |
| smu534 | GCA_020531635.1 | Japan | | HOMD |
| smu535 | GCA_020531605.1 | Japan | | HOMD |
| smu536 | GCA_020531585.1 | Japan | | HOMD |
| smu537 | GCA_020531565.1 | Japan | | HOMD |
| smu538 | GCA_020530585.1 | Japan | | HOMD |
| smu539 | GCA_020530665.1 | Japan | | HOMD |
| smu540 | GCA_020530545.1 | Japan | | HOMD |
| smu541 | GCA_020530565.1 | Japan | | HOMD |
| smu542 | GCA_020530605.1 | Japan | | HOMD |
| smu543 | GCA_020531325.1 | Japan | | HOMD |
| smu544 | GCA_020530905.1 | Japan | | HOMD |
| smu545 | GCA_020530865.1 | Japan | | HOMD |
| smu546 | GCA_020530795.1 | Japan | | HOMD |
| smu547 | GCA_020530785.1 | Japan | | HOMD |
| smu548 | GCA_020531485.1 | Japan | | HOMD |
| smu549 | GCA_020531505.1 | Japan | | HOMD |
| smu550 | GCA_020530805.1 | Japan | | HOMD |
| smu551 | GCA_020531545.1 | Japan | | HOMD |
| smu552 | GCA_020531305.1 | Japan | | HOMD |
| smu553 | GCA_020531465.1 | Japan | | HOMD |
| smu554 | GCA_020529795.1 | Japan | | HOMD |
| smu555 | GCA_020530525.1 | Japan | | HOMD |
| smu556 | GCA_020531445.1 | Japan | | HOMD |
| smu557 | GCA_020531525.1 | Japan | | HOMD |
| smu558 | GCA_020531205.1 | Japan | | HOMD |
| smu559 | GCA_020530925.1 | Japan | | HOMD |
| smu560 | GCA_020529565.1 | Japan | | HOMD |
| smu561 | GCA_020529765.1 | Japan | | HOMD |
| smu562 | GCA_020530465.1 | Japan | | HOMD |
| smu563 | GCA_020530875.1 | Japan | | HOMD |
| smu564 | GCA_020529845.1 | Japan | | HOMD |
| smu565 | GCA_020529825.1 | Japan | | HOMD |
| smu566 | GCA_020531225.1 | Japan | | HOMD |
| smu567 | GCA_020529525.1 | Japan | | HOMD |
| smu568 | GCA_020529785.1 | Japan | | HOMD |
| smu569 | GCA_020529725.1 | Japan | | HOMD |
| smu570 | GCA_020530445.1 | Japan | | HOMD |
| smu571 | GCA_020529485.1 | Japan | | HOMD |
| smu572 | GCA_020530745.1 | Japan | | HOMD |
| smu573 | GCA_020530495.1 | Japan | | HOMD |
| smu574 | GCA_020531245.1 | Japan | | HOMD |
| smu575 | GCA_020530485.1 | Japan | | HOMD |
| smu576 | GCA_020529885.1 | Japan | | HOMD |
| smu577 | GCA_020529665.1 | Japan | | HOMD |
| smu578 | GCA_020529745.1 | Japan | | HOMD |
| smu579 | GCA_020529465.1 | Japan | | HOMD |
| smu580 | GCA_020531285.1 | Japan | | HOMD |
| smu581 | GCA_020529495.1 | Japan | | HOMD |
| smu582 | GCA_020529865.1 | Japan | | HOMD |
| smu583 | GCA_020529595.1 | Japan | | HOMD |
| smu584 | GCA_020530725.1 | Japan | | HOMD |
| smu585 | GCA_020529905.1 | Japan | | HOMD |
| smu586 | GCA_020530705.1 | Japan | | HOMD |
| smu587 | GCA_020530985.1 | Japan | | HOMD |
| smu588 | GCA_020530765.1 | Japan | | HOMD |
| smu589 | GCA_020529645.1 | Japan | | HOMD |
| smu590 | GCA_020529585.1 | Japan | | HOMD |
| smu591 | GCA_020529625.1 | Japan | | HOMD |
| smu592 | GCA_020529545.1 | Japan | | HOMD |
| smu593 | GCA_020531425.1 | Japan | | HOMD |
| smu594 | GCA_020531405.1 | Japan | | HOMD |
| smu595 | GCA_020531165.1 | Japan | | HOMD |
| smu596 | GCA_020530965.1 | Japan | | HOMD |
| smu597 | GCA_020531185.1 | Japan | | HOMD |
| smu598 | GCA_020531105.1 | Japan | | HOMD |
| smu599 | GCA_020531145.1 | Japan | | HOMD |
| smu600 | GCA_020531355.1 | Japan | | HOMD |
| smu601 | GCA_020531065.1 | Japan | | HOMD |
| smu602 | GCA_020530945.1 | Japan | | HOMD |
| smu603 | GCA_020531005.1 | Japan | | HOMD |
| smu604 | GCA_020530125.1 | Japan | | HOMD |
| smu605 | GCA_020531045.1 | Japan | | HOMD |
| smu606 | GCA_020531025.1 | Japan | | HOMD |
| smu607 | GCA_020530165.1 | Japan | | HOMD |
| smu608 | GCA_020530075.1 | Japan | | HOMD |
| smu609 | GCA_020530245.1 | Japan | | HOMD |
| smu610 | GCA_020530185.1 | Japan | | HOMD |
| smu611 | GCA_020530145.1 | Japan | | HOMD |
| smu612 | GCA_020531345.1 | Japan | | HOMD |
| smu613 | GCA_020530625.1 | Japan | | HOMD |
| smu614 | GCA_020529275.1 | Japan | | HOMD |
| smu615 | GCA_020530425.1 | Japan | | HOMD |
| smu616 | GCA_020530105.1 | Japan | | HOMD |
| smu617 | GCA_020530025.1 | Japan | | HOMD |
| smu618 | GCA_020530065.1 | Japan | | HOMD |
| smu619 | GCA_020530305.1 | Japan | | HOMD |
| smu620 | GCA_020530045.1 | Japan | | HOMD |
| smu621 | GCA_020530345.1 | Japan | | HOMD |
| smu622 | GCA_020531385.1 | Japan | | HOMD |
| smu623 | GCA_020530355.1 | Japan | | HOMD |
| smu624 | GCA_020530285.1 | Japan | | HOMD |
| smu625 | GCA_020529345.1 | Japan | | HOMD |
| smu626 | GCA_020530405.1 | Japan | | HOMD |
| smu627 | GCA_020531255.1 | Japan | | HOMD |
| smu628 | GCA_020530365.1 | Japan | | HOMD |
| smu629 | GCA_020529365.1 | Japan | | HOMD |
| smu630 | GCA_020530225.1 | Japan | | HOMD |
| smu631 | GCA_020530325.1 | Japan | | HOMD |
| smu632 | GCA_020530255.1 | Japan | | HOMD |
| smu633 | GCA_020530205.1 | Japan | | HOMD |
| smu634 | GCA_020529985.1 | Japan | | HOMD |
| smu635 | GCA_020529415.1 | Japan | | HOMD |
| smu636 | GCA_003691695.1 | Canada | | HOMD |
| smu637 | GCA_002995555.1 | USA | | HOMD |
| smu638 | GCA_008831325.1 | Brazil | | HOMD |
| smu639 | GCA_009738105.1 | USA | | HOMD |
| smu640 | GCA_900475095.1 | United Kingdom | | HOMD |
| smu641 | GCA_900636835.1 | United Kingdom | | HOMD |
| smu642 | GCA_900638045.1 | United Kingdom | | HOMD |
| smu643 | GCA_900459345.1 | United Kingdom | | HOMD |
| smu644 | GCA_011765545.1 | Brazil | | HOMD |
| smu645 | GCA_011765525.1 | Brazil | | HOMD |
| smu646 | GCA_002213065.1 | China | | HOMD |
| smu647 | GCA_002213035.1 | China | | HOMD |
| smu648 | GCA_002212965.1 | China | | HOMD |
| smu649 | GCA_002212855.1 | China | | HOMD |
| smu650 | GCA_002213005.1 | China | | HOMD |
| smu651 | GCA_002212885.1 | China | | HOMD |
| smu652 | GCA_002212905.1 | China | | HOMD |
| smu653 | GCA_002212845.1 | China | | HOMD |
| smu654 | GCA_002212995.1 | China | | HOMD |
| smu655 | GCA_002212925.1 | China | | HOMD |
| smu656 | GCA_002212935.1 | China | | HOMD |
| smu657 | GCA_011765505.1 | Brazil | | HOMD |
| smu658 | GCA_023109585.1 | USA | | HOMD |
| smu659 | GCA_011765485.1 | Brazil | | HOMD |
| smu660 | GCA_018588785.1 | USA | | HOMD |
| smu661 | GCA_018588765.1 | USA | | HOMD |
| smu662 | GCA_008831345.1 | Netherlands | | HOMD |
| smu663 | GCA_008831365.1 | USA | | HOMD |
| smu664 | GCA_012641085.1 | USA | | HOMD |
| smu665 | GCA_018588825.1 | USA | | HOMD |
| smu666 | GCA_012642405.1 | USA | | HOMD |
| smu667 | GCA_012642225.1 | USA | | HOMD |
| smu668 | GCA_012641665.1 | USA | | HOMD |
| smu669 | GCA_012641625.1 | USA | | HOMD |
| smu670 | GCA_012641605.1 | USA | | HOMD |
| smu671 | GCA_012641615.1 | USA | | HOMD |
| smu672 | GCA_012641575.1 | USA | | HOMD |
| smu673 | GCA_012641565.1 | USA | | HOMD |
| smu674 | GCA_012642205.1 | USA | | HOMD |
| smu675 | GCA_012642185.1 | USA | | HOMD |
| smu676 | GCA_012642165.1 | USA | | HOMD |
| smu677 | GCA_012642365.1 | USA | | HOMD |
| smu678 | GCA_012642145.1 | USA | | HOMD |
| smu679 | GCA_012642125.1 | USA | | HOMD |
| smu680 | GCA_012642105.1 | USA | | HOMD |
| smu681 | GCA_012641545.1 | USA | | HOMD |
| smu682 | GCA_012642085.1 | USA | | HOMD |
| smu683 | GCA_012642055.1 | USA | | HOMD |
| smu684 | GCA_012642035.1 | USA | | HOMD |
| smu685 | GCA_012642015.1 | USA | | HOMD |
| smu686 | GCA_012642005.1 | USA | | HOMD |
| smu687 | GCA_012641975.1 | USA | | HOMD |
| smu688 | GCA_012642345.1 | USA | | HOMD |
| smu689 | GCA_012641965.1 | USA | | HOMD |
| smu690 | GCA_012641935.1 | USA | | HOMD |
| smu691 | GCA_012641925.1 | USA | | HOMD |
| smu692 | GCA_012641905.1 | USA | | HOMD |
| smu693 | GCA_012641855.1 | USA | | HOMD |
| smu694 | GCA_012641885.1 | USA | | HOMD |
| smu695 | GCA_012641825.1 | USA | | HOMD |
| smu696 | GCA_012641845.1 | USA | | HOMD |
| smu697 | GCA_012641765.1 | USA | | HOMD |
| smu698 | GCA_012641775.1 | USA | | HOMD |
| smu699 | GCA_012642355.1 | USA | | HOMD |
| smu700 | GCA_012641805.1 | USA | | HOMD |
| smu701 | GCA_012642325.1 | USA | | HOMD |
| smu702 | GCA_012642305.1 | USA | | HOMD |
| smu703 | GCA_012642275.1 | USA | | HOMD |
| smu704 | GCA_012642265.1 | USA | | HOMD |
| smu705 | GCA_012642235.1 | USA | | HOMD |
| smu706 | GCA_000228765.2 | Brazil | | HOMD |
| smu707 | GCA_000228805.2 | Brazil | | HOMD |
| smu708 | GCA_000229225.2 | Brazil | | HOMD |
| smu709 | GCA_000229345.2 | Brazil | | HOMD |
| smu710 | GCA_000229545.2 | Brazil | | HOMD |
| smu711 | GCA_046035035.1 | Turkey | | Ucuncu MY |
| smu712 | GCA_046035075.1 | Turkey | | Ucuncu MY |
| smu713 | GCA_046035095.1 | Turkey | | Ucuncu MY |
| smu714 | GCA_046035115.1 | Turkey | | Ucuncu MY |
| smu715 | GCA_046035135.1 | Turkey | | Ucuncu MY |
| smu716 | GCA_046035155.1 | Turkey | | Ucuncu MY |
| smu717 | GCA_046035175.1 | Turkey | | Ucuncu MY |
| smu718 | GCA_046035195.1 | Turkey | | Ucuncu MY |
| smu719 | GCA_046035215.1 | Turkey | | Ucuncu MY |
| smu720 | GCA_046035235.1 | Turkey | | Ucuncu MY |
| smu721 | GCA_046035255.1 | Turkey | | Ucuncu MY |
| smu722 | GCA_046035275.1 | Turkey | | Ucuncu MY |
| smu723 | GCA_046035295.1 | Turkey | | Ucuncu MY |
| smu724 | GCA_046035315.1 | Turkey | | Ucuncu MY |
| smu725 | GCA_046035335.1 | Turkey | | Ucuncu MY |
| smu726 | GCA_046035345.1 | Turkey | | Ucuncu MY |
| smu727 | GCA_046035355.1 | Turkey | | Ucuncu MY |
| smu728 | GCA_046035395.1 | Turkey | | Ucuncu MY |
| smu729 | GCA_046035415.1 | Turkey | | Ucuncu MY |
| smu730 | GCA_046035455.1 | Turkey | | Ucuncu MY |
| smu731 | GCA_046036895.1 | Turkey | | Ucuncu MY |
| smu732 | GCA_046036915.1 | Turkey | | Ucuncu MY |
| smu733 | GCA_046036935.1 | Turkey | | Ucuncu MY |
| smu734 | GCA_046036975.1 | Turkey | | Ucuncu MY |
| smu735 | GCA_046037035.1 | Turkey | | Ucuncu MY |

**Table S2. Distribution of CRISPR-Cas subtypes and per-strain spacer counts in the *Streptococcus mutans* dataset.**

| Subtype | mean_spacer_num | sd_spacer_num | n_strains | percentage |
| --- | --- | --- | --- | --- |
| I-C | 36.2 | 26.1 | 141 | 25.730% |
| I-E | 28.3 | 15.9 | 140 | 25.547% |
| II-A | 21.1 | 14.1 | 203 | 37.044% |
| II-C | 13.8 | 11.9 | 63 | 11.496% |
| III-A | 33 | NA | 1 | 0.182% |

**Table S3. Summary of CRISPR spacer matches to bacteriophages, including detected (database-derived), reference phages and UViG from IMGVR.**

| Detected bacteriophages | Spacer Hits | Strain Num | Accession Number |
| --- | --- | --- | --- |
| *Caudoviricetes* sp. isolate ctNo011 | 567 | 309 | BK034220.1 |
| *Caudoviricetes* sp. isolate ctQS92 | 128 | 105 | BK037272.1 |
| *Bacteriophage* sp. isolate 2092_33522 | 6 | 6 | OP076492.1 |
| *Caudoviricetes* sp. isolate ctZwz14 | 4 | 4 | BK043112.1 |
| *Caudoviricetes* sp. isolate ctZxy9 | 4 | 4 | BK023187.1 |
| *Bacteriophage* sp. isolate 0510_49728 | 3 | 3 | OP073292.1 |
| *Bacteriophage* sp. isolate 0803_73704 | 3 | 3 | OP073473.1 |
| *Bacteriophage* sp. isolate 0809_20563 | 3 | 3 | OP073479.1 |
| *Bacteriophage* sp. isolate 1024_16718 | 3 | 3 | OP073633.1 |
| *Bacteriophage* sp. isolate 1180_88491 | 3 | 3 | OP073738.1 |
| *Bacteriophage* sp. isolate 2533_53993 | 3 | 3 | OP074677.1 |
| *Bacteriophage* sp. isolate 2946_18154 | 3 | 3 | OP075063.1 |
| *Bacteriophage* sp. isolate 3473_104527 | 3 | 3 | OP075531.1 |
| *Caudoviricetes* sp. isolate ct70N1 | 3 | 3 | BK041617.1 |
| *Caudoviricetes* sp. isolate ct7cL13 | 3 | 3 | BK041671.1 |
| *Caudoviricetes* sp. isolate ct86y19 | 3 | 3 | BK054982.1 |
| *Caudoviricetes* sp. isolate ctfP217 | 3 | 3 | BK034254.1 |
| *Caudoviricetes* sp. isolate ctJj81 | 3 | 3 | BK028666.1 |
| *Caudoviricetes* sp. isolate ctlX62 | 3 | 3 | BK057302.1 |
| *Caudoviricetes* sp. isolate ctMw715 | 3 | 3 | BK041585.1 |
| *Caudoviricetes* sp. isolate ctQxQ2 | 3 | 3 | BK025256.1 |
| *Caudoviricetes* sp. isolate ctW2I2 | 3 | 3 | BK050645.1 |
| *Caudoviricetes* sp. isolate ctwNN12 | 3 | 3 | BK034841.1 |
| *Bacteriophage* sp. isolate ctb0J3 | 2 | 2 | BK056786.1 |
| *Caudoviricetes* sp. isolate ct5xJ1 | 2 | 2 | BK033907.1 |
| *Caudoviricetes* sp. isolate ctBQ57 | 2 | 2 | BK042058.1 |
| *Caudoviricetes* sp. isolate ctEYj1 | 2 | 2 | BK043174.1 |
| *Caudoviricetes* sp. isolate ctRSn6 | 2 | 2 | BK051431.1 |
| *Bacteriophage* sp. isolate 1820_41452 | 1 | 1 | BK019637.1 |
| *Bacteriophage* sp. isolate 2037_19109 | 1 | 1 | OP074252.1 |
| *Bacteriophage* sp. isolate ct7LD3 | 1 | 1 | OP076432.1 |
| *Caudoviricetes* sp. isolate ct2go1 | 1 | 1 | BK041794.1 |
| *Caudoviricetes* sp. isolate ct2tc2 | 2 | 1 | BK049329.1 |
| *Caudoviricetes* sp. isolate ct3JT3 | 1 | 1 | BK034159.1 |
| *Caudoviricetes* sp. isolate ct95k2 | 1 | 1 | BK049350.1 |
| *Caudoviricetes* sp. isolate ctAZo8 | 1 | 1 | BK041193.1 |
| *Caudoviricetes* sp. isolate ctb7c11 | 1 | 1 | BK041909.1 |
| *Caudoviricetes* sp. isolate ctBV31 | 1 | 1 | BK023171.1 |
| *Caudoviricetes* sp. isolate ctCKp31 | 1 | 1 | BK040271.1 |
| *Caudoviricetes* sp. isolate cthbV15 | 1 | 1 | BK046003.1 |
| *Caudoviricetes* sp. isolate ctHny17 | 1 | 1 | BK034498.1 |
| *Caudoviricetes* sp. isolate cti2V2 | 1 | 1 | BK055991.1 |
| *Caudoviricetes* sp. isolate ctMKS8 | 1 | 1 | BK033526.1 |
| *Caudoviricetes* sp. isolate ctOjW10 | 1 | 1 | BK039719.1 |
| *Caudoviricetes* sp. isolate ctpNf4 | 1 | 1 | BK033341.1 |
| *Caudoviricetes* sp. isolate ctqk65 | 1 | 1 | BK024723.1 |
| *Caudoviricetes* sp. isolate ctUIM5 | 1 | 1 | BK040213.1 |
| *Caudoviricetes* sp. isolate ctVyw6 | 1 | 1 | BK057568.1 |
| *Caudoviricetes* sp. isolate ctWWl1 | 1 | 1 | BK054001.1 |
| *Caudoviricetes* sp. isolate ctx1W1 | 1 | 1 | BK023021.1 |
| *Caudoviricetes* sp. isolate ctXxE1 | 1 | 1 | BK054429.1 |
| *Siphoviridae* sp. ctNqI2 | 1 | 1 | BK014689.1 |

| Reference *Bacteriophage* | Spacer Hits | Strain Num | Accession Number |
| --- | --- | --- | --- |
| *Streptococcus* phage phiKSM96 | 562 | 285 | OQ627164.1 |
| *Streptococcus* phage M102AD | 280 | 183 | NC_028984.1 |
| *Streptococcus* phage M102 | 260 | 176 | NC_012884.1 |
| *Streptococcus* phage smHBZ8 | 224 | 157 | MT430910.1 |
| *Streptococcus* phage APCM01 | 220 | 142 | NC_029030.1 |
| *Streptococcus* phage T12 | 17 | 12 | KM289195.1 |
| *Streptococcus* phage Javan74 | 6 | 3 | MK449005.1 |
| *Streptococcus* phage Javan59 | 4 | 2 | MK448817.1 |
| *Enterococcus* phage vB_EfaS_IME197 | 2 | 2 | KT945994.2 |
| *Staphylococcus* phage PhiSepi-HH1 | 2 | 1 | MT880870.1 |
| *Streptococcus* phage IPP23 | 2 | 2 | KY065464.1 |
| *Streptococcus* phage IPP28 | 2 | 2 | KY065469.1 |
| *Streptococcus* phage IPP43 | 2 | 2 | KY065483.1 |
| *Lactococcus* phage 38502 | 1 | 1 | KX160204.1 |
| *Lactococcus* phage 58502 | 1 | 1 | KX160209.1 |
| *Lactococcus* phage 62503 | 1 | 1 | NC_049811.1 |
| *Lactococcus* phage P1045 | 1 | 1 | NC_049812.1 |
| *Staphylococcus* phage CNPH82 | 1 | 1 | NC_008722.1 |
| *Staphylococcus* phage CNPx | 1 | 1 | NC_031241.1 |
| *Staphylococcus* phage IME1348_01 | 1 | 1 | NC_055036.1 |
| *Staphylococcus* phage PH15 | 1 | 1 | NC_008723.1 |
| *Staphylococcus* phage vB_SepiS-phiIPLA5 | 1 | 1 | NC_018281.1 |
| *Staphylococcus* phage vB_SepiS-phiIPLA7 | 1 | 1 | NC_018284.1 |
| *Staphylococcus* phage vB_SepS_BE20 | 1 | 1 | OQ355699.1 |
| *Staphylococcus* virus 456 | 1 | 1 | MT596497.1 |
| *Staphylococcus* virus BESEP1 | 1 | 1 | MT596498.1 |
| *Staphylococcus* virus vB_SepS_27 | 1 | 1 | MW364971.1 |
| *Staphylococcus* virus vB_SepS_E72 | 1 | 1 | MW364975.1 |
| *Streptococcus* phage 7201 | 1 | 1 | AF145054.1 |
| *Streptococcus* phage A1 | 1 | 1 | MW495853.1 |
| *Streptococcus* phage A25 | 1 | 1 | NC_028697.1 |
| *Streptococcus* phage Javan115 | 1 | 1 | MK448671.1 |
| *Streptococcus* phage Javan406 | 1 | 1 | MK448930.1 |
| *Streptococcus* phage Javan88 | 1 | 1 | MK449009.1 |
| *Streptococcus* phage Javan91 | 1 | 1 | MK448834.1 |
| *Streptococcus* phage phiNJ2 | 1 | 1 | JX879087.1 |
| *Streptococcus* phage Str01 | 1 | 1 | KY349816.1 |
| UNVERIFIED: *Staphylococcus* phage PG-2021_90 | 1 | 1 | MZ417351.1 |
| UNVERIFIED: *Staphylococcus* phage PG-2021_91 | 1 | 1 | MZ417352.1 |
| UNVERIFIED: *Staphylococcus* phage PG-2021_93 | 1 | 1 | MZ417353.1 |

| UViG_ID | Spacer Hits | Strain Num | Phage_UViG |
| --- | --- | --- | --- |
| IMGVR_UViG_1 | 188 | 135 | IMGVR_UViG_2558860320_000002 |
| IMGVR_UViG_2 | 77 | 67 | IMGVR_UViG_2558860300_000002 |
| IMGVR_UViG_3 | 40 | 39 | IMGVR_UViG_2558860317_000003 |
| IMGVR_UViG_4 | 39 | 37 | IMGVR_UViG_2558860300_000001 |
| IMGVR_UViG_5 | 19 | 17 | IMGVR_UViG_2558860317_000004 |
| IMGVR_UViG_6 | 5 | 5 | IMGVR_UViG_3300010283_000408 |
| IMGVR_UViG_7 | 2 | 2 | IMGVR_UViG_2823520609_000003 |
| IMGVR_UViG_8 | 2 | 2 | IMGVR_UViG_2826221612_000001 |
| IMGVR_UViG_9 | 2 | 2 | IMGVR_UViG_3300008679_000212 |
| IMGVR_UViG_10 | 1 | 1 | IMGVR_UViG_2551306247_000001 |
| IMGVR_UViG_11 | 1 | 1 | IMGVR_UViG_2579779011_000001 |
| IMGVR_UViG_12 | 1 | 1 | IMGVR_UViG_2606217751_000005 |
| IMGVR_UViG_13 | 1 | 1 | IMGVR_UViG_2737471995_000004 |
| IMGVR_UViG_14 | 1 | 1 | IMGVR_UViG_2737471996_000001 |
| IMGVR_UViG_15 | 1 | 1 | IMGVR_UViG_2737471999_000001 |
| IMGVR_UViG_16 | 1 | 1 | IMGVR_UViG_3300007499_000039 |
| IMGVR_UViG_17 | 1 | 1 | IMGVR_UViG_3300007500_000151 |
| IMGVR_UViG_18 | 1 | 1 | IMGVR_UViG_3300045988_188298 |

**Table S4.** **Gene repertoire comparison across *Streptococcus mutans* subgroups with CRISPR spacer evidence targeting phages M102, phiKSM96, and smHBZ8.**

| GROUP | M102 | phiKSM96 | | smHBZ8 | |
| --- | --- | --- | --- | --- | --- |
| GENE | *Abhydrolase_8* | *PDDEXK_4* | *Proton_antipo_C* | *MutL* | *CbiN* |
|  |  | *OMP_b-brl_3* | *TniB* | *Peptidase_M26_N* | *Dehydratase_LU* |
|  |  | *TnpB_IS66* | *Tn7_TnsC_Int* | *DUF3278* | *Dehydratase_SU* |
|  |  | *Thioredoxin_2* | *DndE* | *RAMPs* | *DDR* |
|  |  | *Thioredoxin_8* | *DUF4400* | *Ntox44* | *CbiK* |
|  |  | *Cyclophil_like* | *DUF1290* | *CbiC* | *Glyco_transf_21* |
|  |  | *YadA_stalk* | *DUF1028* | *YcxB* | *HutD* |
|  |  | *YadA_head* | *PG_binding_2* | *Dehydratase_MU* | *Pyr_excise* |
|  |  | *YadA_anchor* | *Acetyltransf_6* | *DUF1911* | *MpPF26* |
|  |  | *Arr-ms* | *SmpA_OmlA* | *RimK* | *FTCD_C* |
|  |  | *NeuB* | *YojJ* | *Toxin-deaminase* | *FTCD_N* |
|  |  | *Tox-GHH* | *DUF1850* | *DUF1851* | *FTCD* |
|  |  | *YusW* | *MoaF* | *GAD-like* | *DUF4299* |
|  |  | *DUF2651* | *SnoaL* | *DUF4284* | *CbiD* |
|  |  | *SBBP* | *Vsr* | *WHH* | *CbiG_mid* |
|  |  | *DUF3923* | *Acetyltransf_8* | *DUF3520* | *CbiG_N* |
|  |  | *DUF1351* | *ARD* | *DUF1846* | *CbiG_C* |
|  |  | *DNA_pol_B* | *DUF1343* | *vWF_A* | *Ferric_reduct* |
|  |  | *TspO_MBR* | *Holin_BhlA* | *PilM_2* | *GT-D* |
|  |  | *DUF2231* |  | *PilN* | *Asp4* |
|  |  | *DUF4041* |  | *BetaGal_dom4_5* | *Asp5* |
|  |  | *MUG113* |  | *CRISPR_Cas6* | *DUF4173* |
|  |  | *DUF4362* |  | *zinc_ribbon_15* | *DUF389* |
|  |  | *DUF5381* |  | *DUF4300* | *Glug* |
|  |  | *AP2* |  | *DUF4298* | *PrlF_antitoxin* |
|  |  | *TnsD* |  | *DUF1910* | *Csm2_III-A* |
|  |  | *TniQ* |  | *DUF4336* |  |


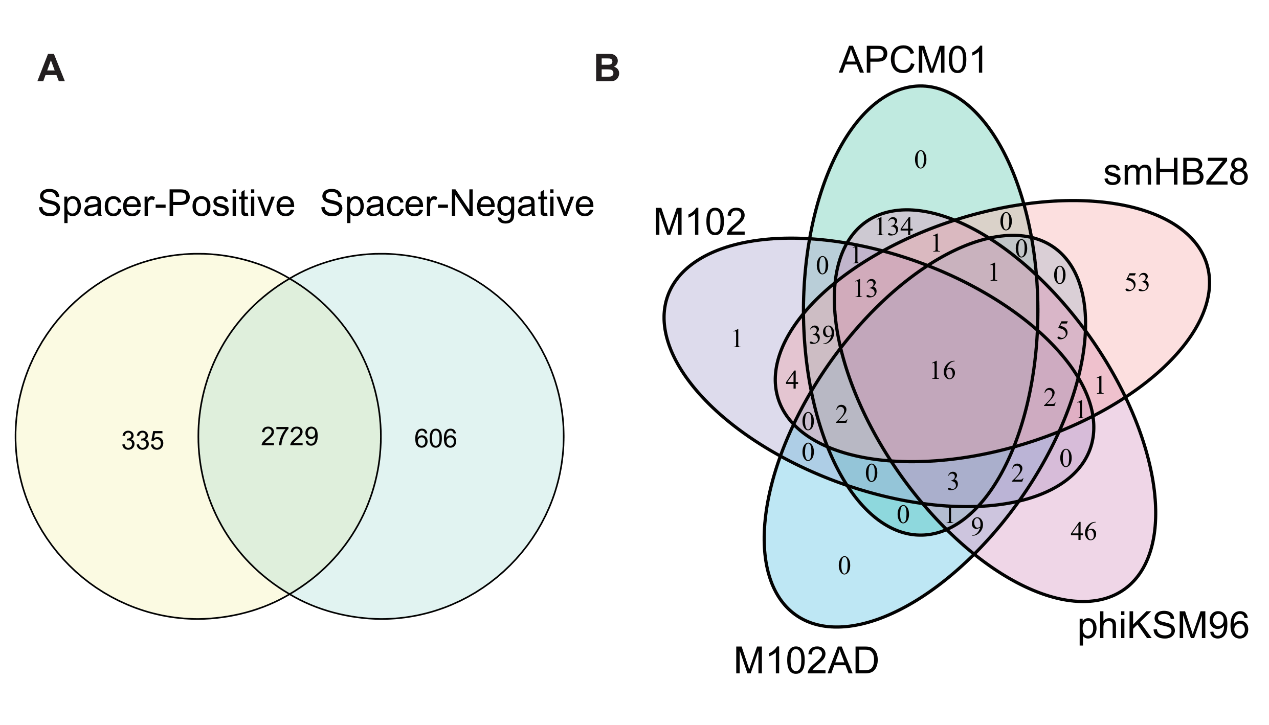


**Figure S1.**

Genomic signatures and host-range overlap of Streptococcus mutans strains based on phage-targeting CRISPR spacer evidence. (A) Distribution of S. mutans genes comparing spacer-positive and spacer-negative groups. (B) Intersection of differential gene sets across five phage-targeting spacer groups.


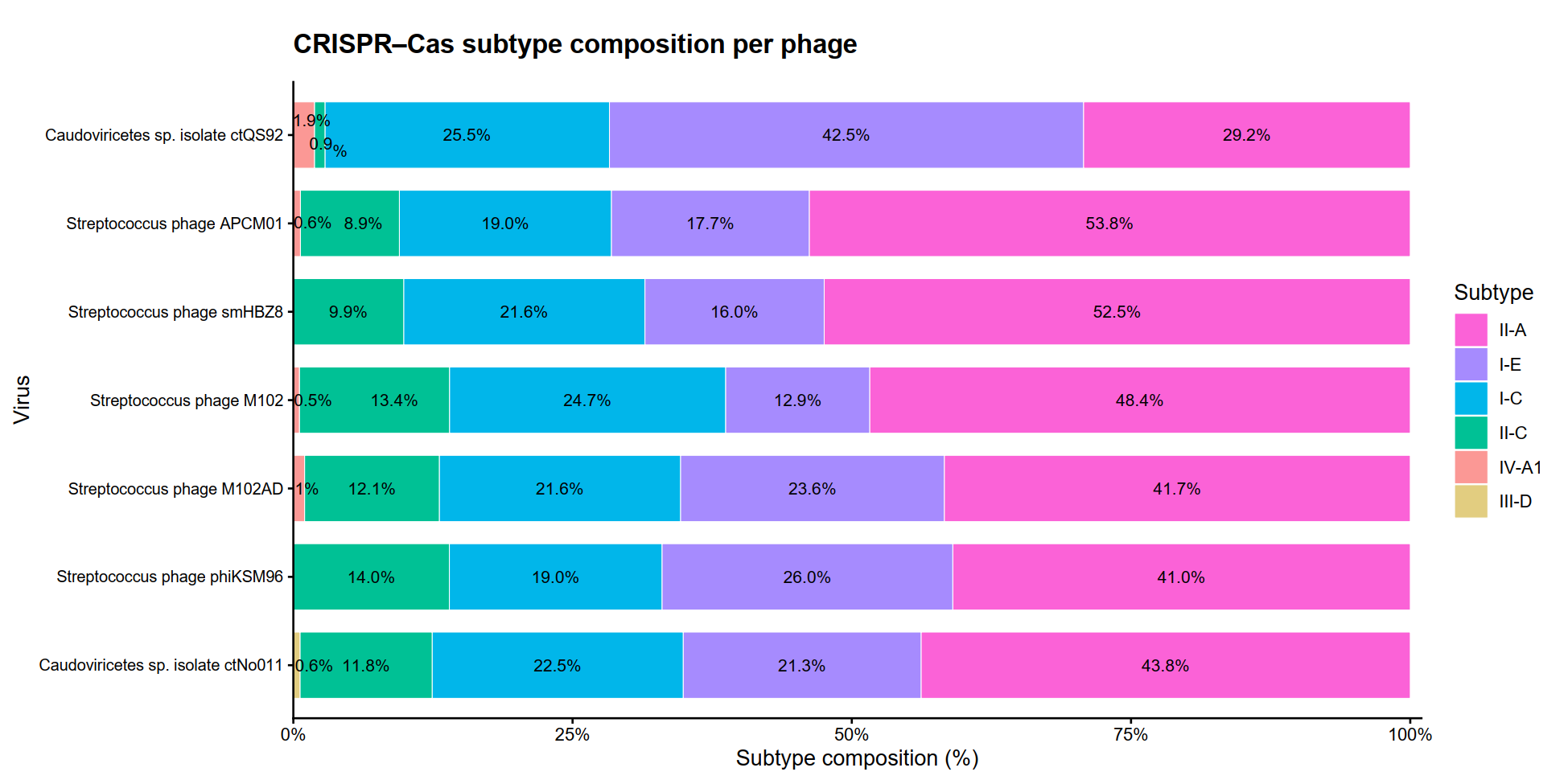


**Figure S2.**

CRISPR-Cas subtype composition of strains targeting each phage (strain-subtype presence).Stacked bars show the percentage of strain-subtype presence events for each phage, where each strain contributes at most once per subtype; percentages sum to 100% within each phage.


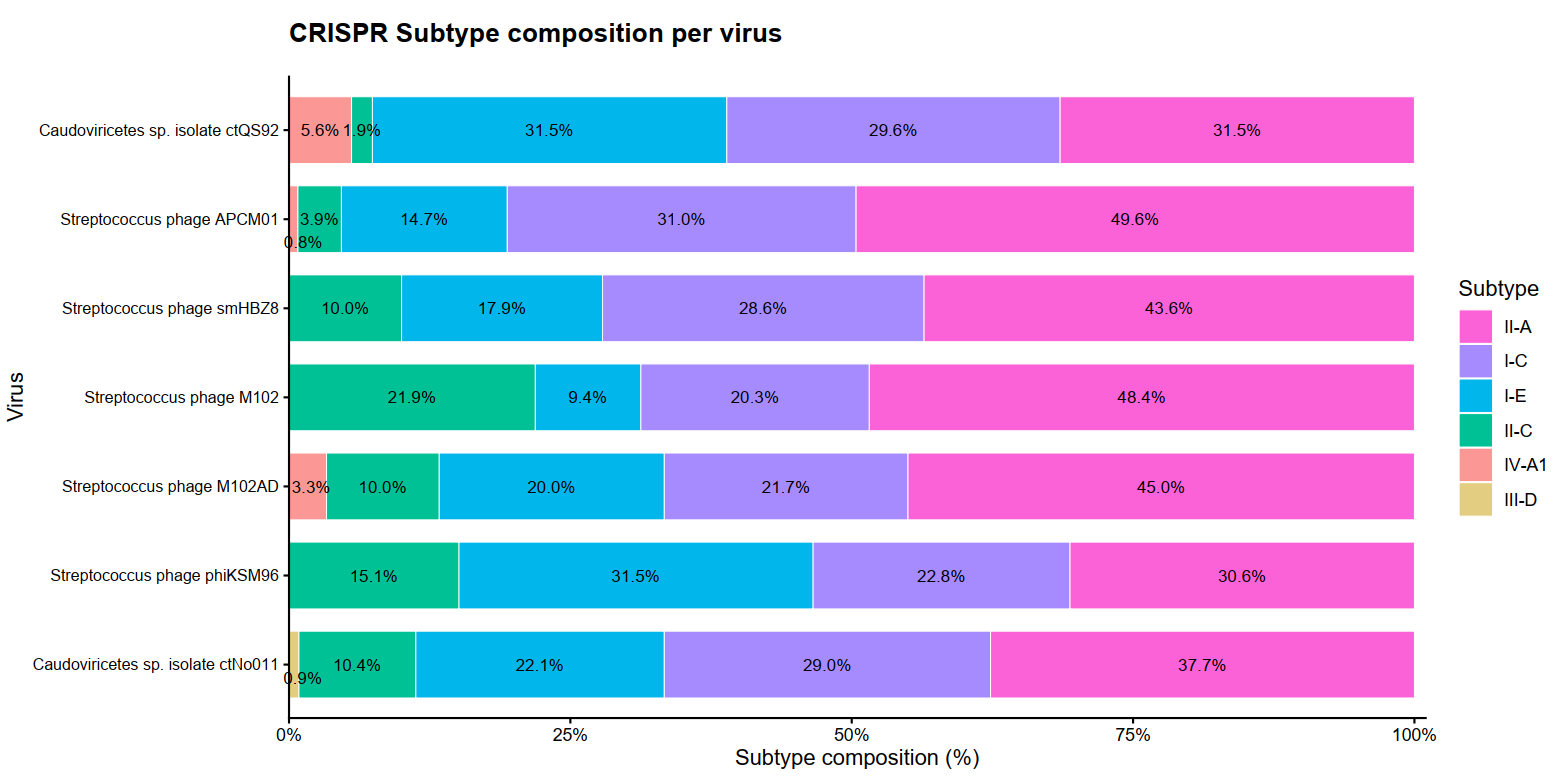


**Figure S3.**

CRISPR-Cas subtype composition of spacer hits per phage (phage-exclusive spacer subset).Stacked bars show the percentage of spacer hits contributed by each CRISPR-Cas subtype for each phage, calculated from the phage-exclusive spacer subset (spacers that matched only one phage).
